# Direct stimulation of ERBB2 highlights a novel cytostatic signaling pathway driven by the receptor Thr^701^ phosphorylation

**DOI:** 10.1038/s41598-020-73835-1

**Published:** 2020-10-09

**Authors:** Marco Gaviraghi, Andrea Rabellino, Annapaola Andolfo, Matthias Brand, Chiara Brombin, Paola Bagnato, Giuseppina De Feudis, Andrea Raimondi, Alberta Locatelli, Daniela Tosoni, Davide Mazza, Luca Gianni, Giovanni Tonon, Yosef Yarden, Carlo Tacchetti, Tiziana Daniele

**Affiliations:** 1 Division of Experimental Oncology, Istituto di Ricovero e Cura a Carattere Scientifico (IRCCS) San Raffaele Scientific Institute, via Olgettina 60, 20132 Milan, Italy; 2 Department of Experimental Medicine, University of Genoa, via De Toni 14, 16132 Genoa, Italy; 3 Protein Microsequencing Facility, Istituto di Ricovero e Cura a Carattere Scientifico (IRCCS) San Raffaele Scientific Institute, via Olgettina 60, 20132 Milan, Italy; 4 Experimental Imaging Centre, Istituto di Ricovero e Cura a Carattere Scientifico (IRCCS) San Raffaele Scientific Institute, via Olgettina 58, 20132 Milan, Italy; 5 University Centre for Statistics in the Biomedical Sciences, Vita-Salute San Raffaele University, via Olgettina 58, 20132 Milan, Italy; 6 Department of Oncology, Istituto di Ricovero e Cura a Carattere Scientifico (IRCCS) San Raffaele Scientific Institute, via Olgettina 60, 20132 Milan, Italy; 7 Department of Experimental Oncology, IEO, European Institute of Oncology IRCCS, 20100 Milan, Italy; 8 Center for Translational Genomics and Bioinformatics, Istituto di Ricovero e Cura a Carattere Scientifico (IRCCS) San Raffaele Scientific Institute, via Olgettina 60, 20132 Milan, Italy; 9 Weizmann Institute of Science, 76100 Rehovot, Israel; 10 Vita-Salute San Raffaele University, via Olgettina 58, 20132 Milan, Italy; 11 Present Address: QIMR Berghofer Medical Research Institute, Brisbane, QLD 4029 Australia; 12 Present Address: CeMM Research Center for Molecular Medicine of the Austrian Academy of Sciences, 1090 Vienna, Austria

**Keywords:** Cell signalling, Breast cancer

## Abstract

ERBB2 is a ligand-less tyrosine kinase receptor expressed at very low levels in normal tissues; when overexpressed, it is involved in malignant transformation and tumorigenesis in several carcinomas. In cancer cells, ERBB2 represents the preferred partner of other members of the ERBB receptor family, leading to stronger oncogenic signals, by promoting both ERK and AKT activation. The identification of the specific signaling downstream of ERBB2 has been impaired by the lack of a ligand and of an efficient way to selectively activate the receptor. In this paper, we found that antibodies (Abs) targeting different epitopes on the ERBB2 extracellular domain foster the activation of ERBB2 homodimers, and surprisingly induce a unique cytostatic signaling cascade promoting an ERK-dependent ERBB2 Thr^701^ phosphorylation, leading to AKT de-phosphorylation, via PP2A Ser/Thr phosphatases. Furthermore, the immunophilin Cyclophilin A plays a crucial role in this pathway, acting as a negative modulator of AKT de-phosphorylation, possibly by competing with Ser/Thr phosphatases for binding to AKT. Altogether, our data show that Ab recognizing ERBB2 extracellular domain function as receptor agonists, promoting ERBB2 homodimer activation, leading to an anti-proliferative signaling. Thus, the ultimate outcome of ERBB2 activity might depend on the dimerization status: pro-oncogenic in the hetero-, and anti-oncogenic in the homo-dimeric form.

## Introduction

ERBB2 belongs to the ERBB family of receptor tyrosine kinase (RTK), which comprises also EGF receptor (EGFR or ERBB1 or HER1), ERBB3 (HER3), and ERBB4 (HER4). ERBB2 is expressed at very low levels in normal tissues^1^, but it is overexpressed in breast, ovary, prostate, non-small cell lung cancer and in several other carcinomas, and is involved in malignant transformation and tumorigenesis^2,3^. In particular, around 20% of breast cancers (BrCa) display overexpression of ERBB2 (ERBB2-BrCa), leading to a more aggressive clinical course and a worst outcome^4,5^.

Whereas the specific outcome of ERBB2 activation and its downstream signaling are not yet fully understood, the molecular events induced by ligand stimulation of the prototypical ERBB receptor, i.e. EGFR, have been thoroughly investigated. Ligand binding to the receptor triggers receptor dimerization and the trans-phosphorylation of tyrosine residues in the cytoplasmic domain of the partner receptor^6^. The phosphorylated tyrosine residues represent docking sites for several different adaptor proteins, leading to the activation of downstream signaling pathways, including the PI3kinase (PI3K)/AKT and the RAS/MAPK cascades^7–9^.

Compared to other members of the family, ERBB2 has no known ligand; thus, in physiological conditions its activation depends on heterodimerization with other ERBB family members^10,11^. Because of its structural conformation, displaying an extended dimerization arm at steady state, ERBB2 represents the preferred partner for other ERBBs^12–14^. In a heterodimer context ERBB2 prevents the downregulation of the dimeric receptor, decreasing the rate of its internalization and of ligand release from the partner ERBB, thus potentiating the ERK and AKT signaling cascades^15–19^. Indeed, ERBB2 activation in a heterodimer context has been shown to promote cell survival and breast carcinogenesis^16,20–24^. Accordingly ERBB2 silencing has been reported to induce an arrest in the G1 phase of the cell cycle, primarily as a result of AKT inactivation^25,26^.

The absence of a known ligand for ERBB2 has been the major difficulty to address how the receptor functions in ERBB2-overexpressing BrCa cells, and indeed most studies have relied on the stimulation of the partner receptor in ERBB2-containing heterodimers.

Here, by exploiting two different antibodies targeting ERBB2, we identified a common downstream signaling leading to an ERK-dependent AKT de-phosphorylation. In particular, upon binding to ERBB2, the antibodies stimulate the phosphorylation of ERK, which in turn phosphorylates the Thr^701^ residue of the receptor and triggers AKT inactivation. We report that the immunophilin Cyclophilin A (CyPA) is a master switch of this signaling pathway, as it normally binds to phospho-AKT, protecting it from de-phosphorylation. Upon activation of ERBB2 and consequently of ERK, CyPA translocates onto the receptor cytoplasmic tail, possibly leaving AKT available for the activity of PP2A phosphatases. Furthermore, we show that ERK and CyPA cooperate in a positive feedback loop keeping ERBB2 active.

Altogether, our data demonstrate that antibodies function as agonists for ERBB2, promoting receptor homodimerization and a specific and previously unidentified signaling pathway, counteracting the oncogenic pro-survival effect induced by ERBB2-containing heterodimers.

## Results

### Antibodies against ERBB2 extracellular domain promote AKT de-phosphorylation via ERK

To identify ERBB2-specific contribution in modulating intracellular signaling pathways, we sought to stimulate the receptor with two antibodies (Abs) targeting different epitopes in the ERBB2 extracellular domain (ECD). In particular, we used Pertuzumab (PZ^27^) recognizing domain II, and Trastuzumab (TZ^28^) targeting domain IV of the receptor^29^.

**Figure 1:**
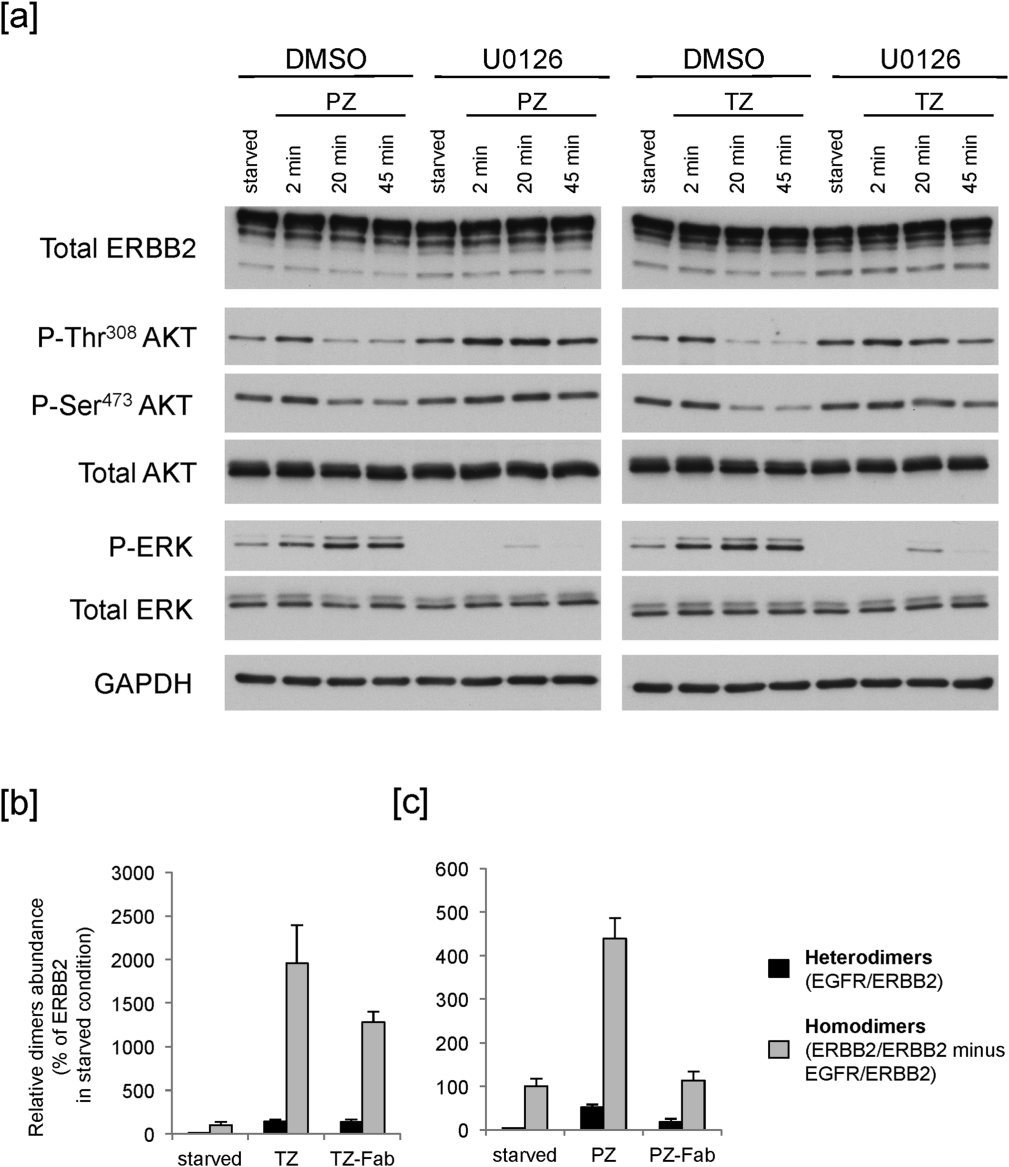
Abs trigger an ERBB2-specific downstream signaling and induce ERBB2 homodimerization. (**a**) SK-Br3 cells were serum-starved for 20 min, in the presence of 10 μM U0126 or vehicle (DMSO), and treated with 20 μg/ml PZ or 10 μg/ml TZ at 37 °C for the indicated times. Lysates were analysed by WB. One experiment is shown as representative of three. Changes in phospho-AKT and phospho-ERK levels are not due to protein degradation, as supported by total AKT and total ERK labelling. The total ERBB2, p-Ser^473^ AKT and GAPDH blots were cropped from the same gel. The p-ERK and p-Thr^308^ AKT blots were cropped from the same gel. The total ERK and total AKT blots were cropped from the same gel. TZ- and PZ-treated samples derive from the same experiment and the gels/blots have been processed in parallel. The images of the full scan western blots are provided in Supplementary Fig. 8. (**b**,**c**) SK-Br3 cells were serum-starved for 20 min at 37 °C, and treated with 10 μg/ml TZ or TZ-Fab (**b**) or with 20 μg/ml PZ or PZ-Fab (**c**) for additional 10 min at 37 °C. Lysates were immunoprecipitated with an ERBB2-specific antibody (starved) and/or an anti-human specific antibody (all samples). Immunoprecipitates were subjected to non-reducing SDS-PAGE and Coomassie staining followed by mass spectrometry. Graphs show mean ± st. dev. from three technical replicates (p < 0.0001 for TZ and TZ-Fab, and p = 0.0003 for PZ, Student's *t* test). For TZ, one experiment is shown as representative of two.

PI3K/AKT survival and RAS/MAPK proliferation pathways are the major signaling pathways activated by ERBB2 heterodimers^30^, thus we initially investigated their phosphorylation kinetics in response to treatments with Abs in SK-Br3 human breast cancer cells. For AKT we focused on the two major phosphorylation residues phosphorylated in response to RTK activation, and necessary for AKT full enzymatic activity, i.e. Thr^308^ and Ser^473^. To avoid long-term signaling adaptation mechanisms, short-term kinetics (2, 20 and 45 min) after treatment with Abs was evaluated. By western blotting (WB) upon treatment with either TZ or PZ, we observed an early persistent increase of phospho-ERK, and a dramatic reduction of the AKT Thr^308^ and Ser^473^ residues phosphorylation, preceded by a transient mild increase (Fig. 1a).

As Abs modulate ERK and AKT in opposite directions, we tested whether the two events are interdependent, evaluating the ERK and AKT phosphorylation state upon treatment with either one of the two Abs in the presence of the MEK1 inhibitor U0126, to prevent ERK activation (Ab/U0126). Ab/U0126 treatment significantly delayed AKT de-phosphorylation of both Thr^308^ and Ser^473^ residues (Fig. 1a), showing that the reduction in phospho-AKT levels in the presence of ERBB2-targeted antibodies depends on ERK activation.

To corroborate these findings, we performed TZ treatment in an additional cell line (BT474) showing ERBB2 levels comparable to SK-Br3 and in MDA-MB-468 cells, which express negligible levels of ERBB2 (Supplementary Fig. 1a). We found that only cells expressing high levels of ERBB2 responded to TZ, showing an ERK-dependent AKT de-phosphorylation (Supplementary Fig. 1b).

As a proof of principle that our findings obtained in SK-Br3, and BT474 cells could be reproduced in primary human ERBB2-BrCa, we established primary cell cultures from patient-derived-xenografts (PDX) developed from one ERBB2-positive patient and one ERBB2-negative BrCa patient, as control. The fraction of cells expressing ERBB2 was assessed by immunofluorescence (IF, data not shown), and only cell cultures obtained from the ERBB2-positive PDX displayed ERBB2 expression in a sizable proportion of the cells (18%).

ERBB2-positive PDX derived cells, but not ERBB2-negative PDX derived cells, responded to TZ and TZ/U0126, displaying an ERK-dependent reduction of the levels of phospho-AKT (Supplementary Fig. 1c), thus supporting the data obtained using SK-Br3, and BT474 immortalized cell lines (Fig. 1a, and Supplementary Fig. 1b).

### Ab-induced AKT de-phosphorylation is an exclusive ERBB2 signaling

As SK-Br3 cells express high ERBB2 levels, detectable levels of EGFR and ERBB3, and very low or no ERBB4 (Supplementary Fig. 1a), we sought to assess whether ERBB2 requires the cooperation of other ERBB family members to respond to Abs. Thus, we silenced SK-Br3 cells for both EGFR and ERBB3 (Supplementary Fig. 2a), and treated them with either Ab or Ab/U0126. In all conditions, cells responded as control cells transfected with a non-targeting (NT) siRNA (Supplementary Fig. 2b), suggesting that EGFR and ERBB3 are dispensable for the response to ERBB2-targeted Abs. We then asked whether stimulation of ERBB1 or ERBB3 would mimic the downstream signaling elicited by Abs. Thus, SK-Br3 cells were treated with EGF (ligand for EGFR), or heregulin β1 (HRG, ligand for ERBB3): in contrast to Abs (Supplementary Fig. 3a), treatment with EGF or HRG promoted both ERK and AKT phosphorylation (Supplementary Fig. 3b or 3c, respectively), suggesting that the ERK-dependent AKT inactivation is an Ab-exclusive signaling.

### Abs binding promotes ERBB2 homodimerization

EGFR and ERBB3 dispensability for the response to the Abs suggest that the ERK-dependent AKT inactivation is likely due to ERBB2 homodimerization.

Based on this hypothesis, we first evaluated the role of the ERBB2 tyrosine kinase activity in the process, by treating SK-Br3 cells with Abs in presence or absence of Lapatinib, an ERBB2 and EGFR tyrosine kinase inhibitor. Under these conditions, Lapatinib completely abrogated the Ab-dependent signaling (Supplementary Fig. 3d), confirming that an active ERBB2 tyrosine kinase is required.

To assess whether Ab binding promotes ERBB2 homodimerization, we performed a mass spectrometry analysis of the ERBB2 containing complexes immunoprecipitated after 10 min of treatment with TZ or PZ (Supplementary Fig. 4 and Supplementary Table S1), and cross-linking with membrane-impermeable DTSSP. WB analysis in not-reducing conditions showed a decrease in the abundance of the monomeric form and an increase of the ERBB2-positive high molecular weight species upon treatment with TZ or PZ (Supplementary Fig. 4d). Peptide analysis revealed that Ab treatment induced a 20-fold (TZ, Fig. 1b) or a five-fold (PZ, Fig. 1c) increase in the abundance of ERBB2-containing dimers.

To rule out the possibility that the higher levels of ERBB2 dimerization might be due to the clustering effect promoted by the bivalent Abs, we generated TZ and PZ Fab fragments (TZ-Fab and PZ-Fab, respectively). By immunoprecipitation (IP) experiments followed by mass spectrometry, we found that, similarly to the bivalent Ab, TZ-Fab promoted a 13-fold increase in the abundance of ERBB2-containing dimers (Fig. 1b). Moreover, by quantifying the fraction of EGFR (to estimate heterodimers) and ERBB2 (to estimate all dimers) engaged in dimers (normalizing the values for their respective amounts in the input fractions), we found that in each condition heterodimers represented approximately 7% of all dimers (Fig. 1b). By contrast, IP experiments followed by mass spectrometry showed that, at odds with the bivalent antibody, PZ-Fab did not induce receptor dimerization, possibly because it engages the dimerization arm of ERBB2 (Fig. 1c).

Altogether, these results support the conclusion that Abs promote the two major events leading to ERBB activation upon ligand binding, i.e. receptor dimerization and activation of a downstream signaling, thus acting as agonists for ERBB2.

### Ab-induced AKT de-phosphorylation depends on Ser/Thr phosphatase activity

There are three main alternative ways to obtain an ERK-dependent reduction of phospho-AKT levels: (i) inhibition of the PI3K/AKT phosphorylation axis; (ii) activation of Ser/Thr phosphatases leading to AKT de-phosphorylation; (iii) a combination of the two.

We first tested the possible contribution of the well-known ERK-dependent inhibition of the PI3K/AKT phosphorylation axis, i.e. PI3K^31,32^, and PTEN^33^ (Supplementary Fig. 5).

Phospho-ERK may control the PI3K-dependent activation of AKT via mTORC1. We assessed the phosphorylation status of the mTORC1 downstream effector p70S6 kinase (S6kinase), as readout of mTORC1 activation^32,34,35^ (Supplementary Fig. 5a), and found no differences in samples treated with TZ or TZ/U0126 (Supplementary Fig. 5c), thus excluding an inhibitory effect on PI3K and subsequently of AKT phosphorylation. Accordingly, SK-Br3 cells treated with either the mTORC1 inhibitor rapamycin, or the PI3kinase inhibitor LY294002, before and during TZ treatment showed a complete inhibition of S6kinase phosphorylation, thus excluding a role for the ERK pathway in controlling mTORC1 activity in this cellular model (Supplementary Fig. 5e).

We then evaluated the possible involvement of PTEN, a cytoplasmic phosphatidylinositol-3,4,5-trisphosphate 3-phosphatase, able to counteract the activity of PI3K, once recruited on the plasma membrane (PM). It has been reported that PTEN recruitment to the PM involves the kinase activity of ERK, the subsequent phosphorylation of MEK1 Thr^292^ residue, and the formation and translocation of a tri-partite complex comprising phosphorylated MEK1, MAGI1 and PTEN to the PM^33^ (Supplementary Fig. 5b). Thus, we analysed the membrane/cytosol partitioning of the three components of the complex and found that it is similar in cells treated with TZ and TZ/U0126 (Supplementary Fig. 5d). In particular, phospho-MEK1 was exclusively present in the cytosolic fraction, MAGI1 in the membrane fraction, and PTEN mainly in the cytosol, excluding also this pathway in the Ab-induced AKT inactivation.

Having excluded a mechanism involving the inhibition of the AKT phosphorylation process, to explain the reduced levels of phospho-AKT promoted by TZ or PZ, we then evaluated the possible contribution of Ser/Thr phosphatase activity. Different phosphatases have been reported to function on AKT specific phospho-residues; in particular, PP2C family members work exclusively on Ser^473^, PP2A holoenzymes on Thr^308^, and PP1 complexes on Thr^450^ and/or Ser^473^^36,37^. Thus, we exploited well-established inhibitors of PP1 and PP2A families of Ser/Thr phosphatases, i.e. calyculin, okadaic acid, and tautomycin, in SK-Br3 cells treated with TZ, either as single agents or in combination. While the phosphorylation levels of AKT Ser^473^ residue were not affected by any of the inhibitor combinations (Supplementary Fig. 6a and 6c), the phosphorylation levels of AKT Thr^308^ residue increased significantly in the presence of higher concentrations of okadaic acid. As the shape of the de-phosphorylation kinetics curves in control and phosphatase inhibitors-treated samples was comparable, we concluded that inhibition of the catalytic activity of Ser/Thr phosphatases per se does not mimic the TZ/U0126 condition (Supplementary Fig. 6a and 6b). By contrast, TZ/U0126 treatment significantly delayed AKT de-phosphorylation kinetics, leading to the possibility that ERK may regulate the association of phosphatases to phospho-AKT.

### Cyclophilin A interacts with phospho-AKT in an ERK-dependent manner

**Figure 2:**
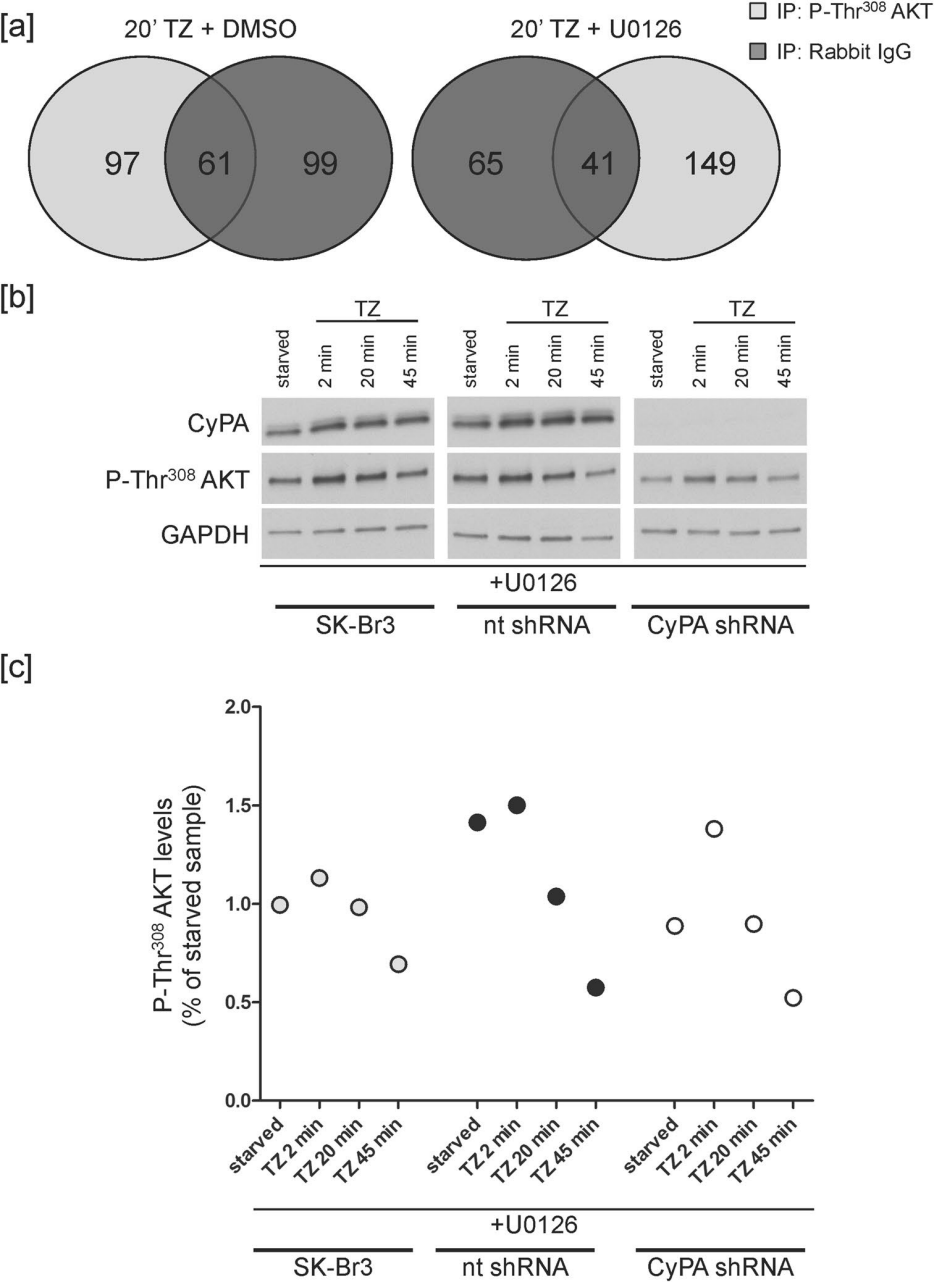
CyPA protects phospho-AKT from de-phosphorylation. (**a**) SK-Br3 cells were serum-starved for 20 min and treated with 10 μg/ml TZ at 37 °C for additional 20 min either in the presence of U0126 or DMSO, as control. Lysates were immunoprecipitated using an anti-phospho-Thr^308^ AKT antibody or rabbit IgG, as control. Venn diagrams compile the comparison of the mass spectrometry results from the phospho-Thr^308^ AKT and rabbit IgG IP in each experimental condition. Numbers indicate proteins identified either specifically in each of the two samples or shared between them. (**b**,**c**) SK-Br3 cells were either not transduced or transduced with a non-targeting (nt) or a CyPA-specific shRNA for 15 days. Cells were serum-starved for 20 min in the presence of the MEK inhibitor U0126, and treated with 10 μg/ml TZ at 37 °C for the indicated times. (**b**) Lysates were analysed by WB. The not transduced (SK-Br3), control (nt) shRNA- or CyPA-specific (CyPA) shRNA-transduced samples derive from the same experiment and the gels/blots have been processed in parallel. The images of the full scan western blots are provided in Supplementary Fig. 9. (**c**) Data points from one experiment are displayed. One experiment is shown as representative of five. A statistically significant difference was found at 20 and 45 min of treatment upon comparison of CyPA-silenced with control SK-Br3 cells (Kruskal Wallis and post-hoc test with Bonferroni correction, *p* = 0.026 and *p* = 0.014, respectively).

To unravel the machinery involved in the regulation of ERBB2-dependent AKT de-phosphorylation, we analysed phospho-AKT interacting proteins by mass spectrometry. Lysates of cells treated for 20 min (time point displaying the greatest difference in phospho-AKT levels between Ab and Ab/U0126, Fig. 1 and Supplementary Fig. 6b) with TZ, or TZ/U0126, were immunoprecipitated using an anti-phospho-Thr^308^ AKT antibody. Proteomic analysis showed that, in the TZ/U0126-treated cells, phospho-AKT interacted, among other proteins, with the Ser/Thr-protein phosphatase 2A (PP2A)-catalytic subunit beta isoform (PPP2CB, UniProtKB accession number: P62714, 5% sequence coverage), and the peptidyl-prolyl cis–trans isomerase A (Cyclophilin A, CyPA) (PPIA, UniProtKB accession number: P62937, 23% sequence coverage) (Fig. 2a and Supplementary Table S2). In the TZ-treated cells, instead, we could not detect any PP2A or CyPA bound to the residual phospho-AKT.

### CyPA silencing promotes AKT de-phosphorylation in the absence of ERK signaling

CyPA is a member of the immunophilin family, which comprises cyclophilins, FK506-binding proteins and parvulins^38^. The best-known member of the parvulin group is Pin1, which has been reported to control AKT phosphorylation and stability^36^.

We found that CyPA is associated to phospho-AKT, suggesting two alternative scenarios, i.e. CyPA exerts a regulatory role on AKT de-phosphorylation or CyPA is a target of AKT activity.

Since it has been described that AKT can phosphorylate CyPA^39^, we tested whether this event occurred in our experimental setting. Thus, we immunoprecipitated CyPA from cells treated with TZ or TZ/U0126, and probed with two antibodies specific for phospho-AKT substrates (RXXS/T* and RXRXXS/T*)^40^ by WB. In neither case we could detect any labelling (Supplementary Fig. 7a), suggesting that in our system phospho-AKT does not act upstream of CyPA, but it is rather a downstream effector of CyPA.

To identify the role of CyPA in the TZ-induced AKT de-phosphorylation, we silenced CyPA expression by transducing SK-Br3 cells with a short hairpin RNA (shRNA) targeting CyPA, and evaluated the AKT de-phosphorylation kinetics upon TZ/U0126 administration (condition in which we found CyPA associated to phospho-AKT, Fig. 2b,c and Supplementary Fig. 7b). In five independent experiments, CyPA silencing consistently impaired the AKT Thr^308^ de-phosphorylation delay observed upon TZ/U0126 treatment (Fig. 2b,c), but not the AKT Thr^308^ de-phosphorylation promoted by TZ (Supplementary Fig. 7c), suggesting that ERK may stimulate AKT de-phosphorylation by promoting the detachment of CyPA from phospho-AKT.

### CyPA is recruited by ERBB2 upon Ab treatment

**Figure 3:**
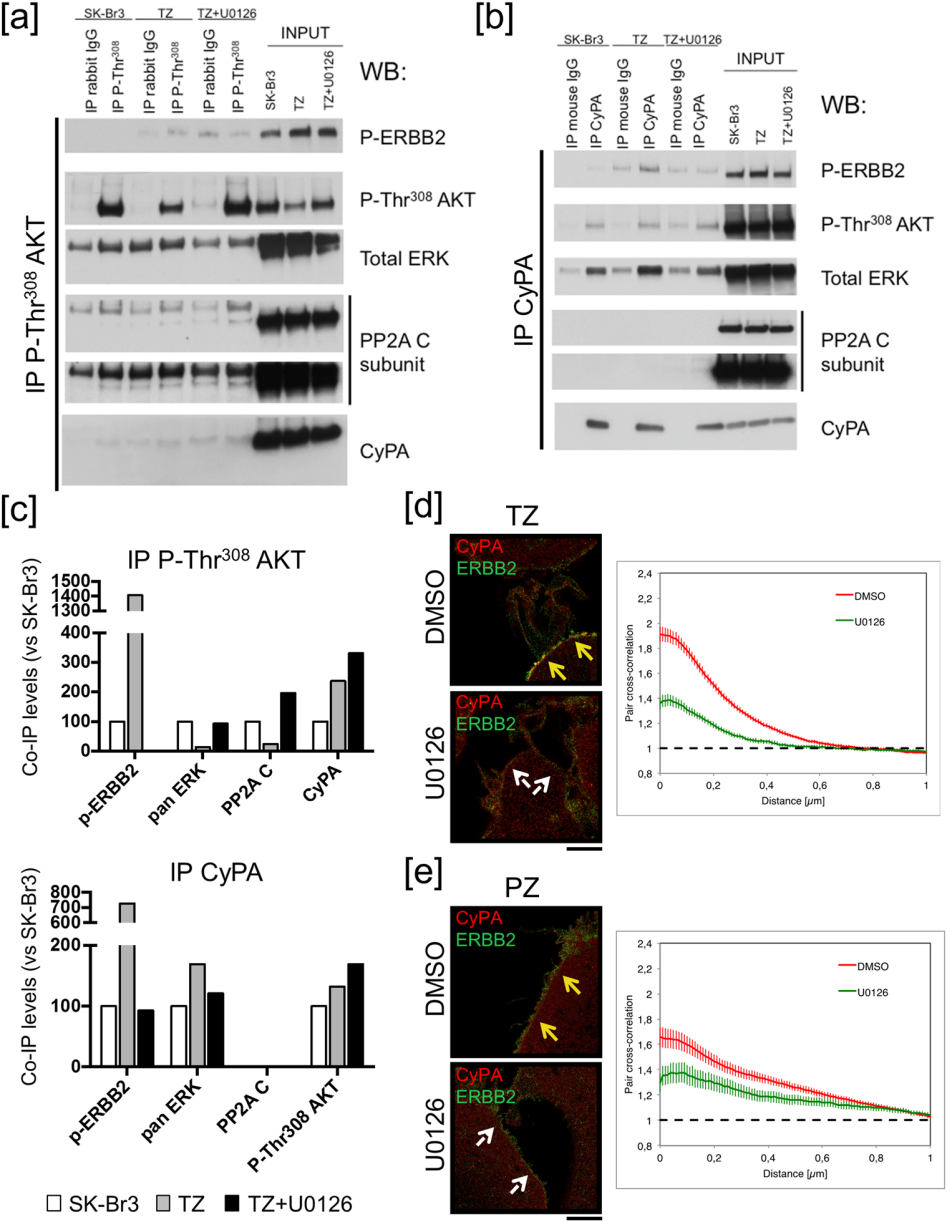
Abs recruit an ERK/CyPA/phospho-AKT signaling hub onto ERBB2. (**a**–**c**) SK-Br3 cells were either left untreated or serum-starved for 20 min, in the presence of 10 μM U0126 or vehicle (DMSO), and treated with 10 μg/ml TZ at 37 °C for 20 min. Lysates were immunoprecipitated with antibodies specific for either P-Thr^308^ AKT (**a**) or CyPA (**b**). Immunoprecipitates were analysed by WB. (**a**,**b**) The images of the full scan western blots are provided in Supplementary Fig. 10. (**c**) Amounts of co-immunoprecipitated (co-IP) proteins were normalized both on the amount of the immunoprecipitated (IP) protein (P-Thr^308^ AKT in **a** and CyPA in **b**) and on their levels in control TZ-untreated SK-Br3 cells. One experiment is shown, as representative of two. Signal in the control IgG lanes in the TZ and TZ + U0126 samples might be due to the direct binding of TZ antibody to Protein G dynabeads, and was subtracted before normalization. The higher molecular weight of bands in the IP, as compared to the input, lanes might be due to the crosslinker. (**d**,**e**) SK-Br3 cells were serum-starved for 20 min, in the presence of 10 μM U0126 or vehicle (DMSO), and treated with 10 μg/ml TZ (**d**) or 20 μg/ml PZ (**e**) at 37 °C for 20 min. On the day of acquisition, cells were processed for immunofluorescence and analysed by dSTORM super-resolution microscopy. Co-localization was assessed by pair cross-correlation analysis. For TZ, ten regions/field were chosen and eleven fields were analysed, out of three independent experiments that were pooled together. Data are presented as mean ± s.e.m. (p < 5 × 10^–9^ upon Kolmogorov–Smirnov test on cross-correlation signal amplitude). For PZ, ten regions/field were chosen and eight fields were analysed, out of two independent experiments that were pooled together. Data are presented as mean ± s.e.m. (p < 0.05 upon Kolmogorov–Smirnov test on cross-correlation signal amplitude). Scale bar: 4 μm.

To better understand the role of CyPA in regulating AKT de-phosphorylation, we explored the landscape of molecular interactions between the members of ERBB2 signaling pathway and CyPA by performing direct and reverse IP experiments. Lysates from SK-Br3 cells either untreated, or treated with TZ or TZ/U0126 for 20 min, were immunoprecipitated with an antibody against phospho-Thr^308^ AKT. We found that in TZ-treated, compared to untreated cells, phospho-AKT co-immunoprecipitated about ten folds higher amounts of the phosphorylated form of ERBB2 (phospho-ERBB2), and about two folds the amount of CyPA, but almost no ERK or PP2A catalytic subunit (possibly because of the low amount of AKT still phosphorylated in this condition); conversely, when TZ/U0126-treated cells were compared to untreated cells, phospho-AKT co-immunoprecipitated equal amounts of ERK, higher amounts of CyPA and PP2A catalytic subunit, but not phospho-ERBB2 (Fig. 3a,c).

The IP using a CyPA-specific antibody confirmed these data, with two noteworthy exceptions: a more efficient precipitation of ERK in TZ-treated as compared to untreated cells (Fig. 3b,c), and the lack of PP2A precipitation in both TZ and TZ/U0126 conditions. Strikingly, we also found that in TZ treated, but not in TZ/U0126 treated cells, CyPA co-immunoprecipitated phospho-ERBB2.

To further support this observation, we investigated the subcellular distribution of CyPA (an abundant cytosolic protein), with respect to the PM-associated ERBB2 receptor, in SK-Br3 cells upon Ab or Ab/U0126 treatment. Cells were analysed by means of direct stochastic optical reconstruction super-resolution microscopy (dSTORM), and co-localization assessed by pair cross-correlation^41^. We found that in Ab, compared to Ab/U0126 condition, the co-localization between CyPA and ERBB2 was significantly higher (Fig. 3d,e), further supporting the finding that ERBB2-targeted Abs induced the recruitment of CyPA onto ERBB2 receptor.

Altogether, these results suggest a model in which Abs induce a phospho-ERK-dependent recruitment of a signaling complex comprising phospho-ERK, CyPA, and phospho-AKT, onto the C-terminal domain of ERBB2. Inhibition of ERK phosphorylation, in turn, leads to an increased interaction between CyPA and phospho-AKT and impairs the recruitment of the complex to the receptor. Because phospho-AKT levels are dramatically reduced upon Ab, but not upon Ab/U0126 treatment, and CyPA appears to play a role in protecting AKT from de-phosphorylation, we hypothesize that CyPA recruitment onto ERBB2 C-terminal domain might release AKT from CyPA, leaving it accessible to Ser/Thr phosphatases (see below ).

### CyPA and PP2A bind to the same AKT residues

**Figure 4:**
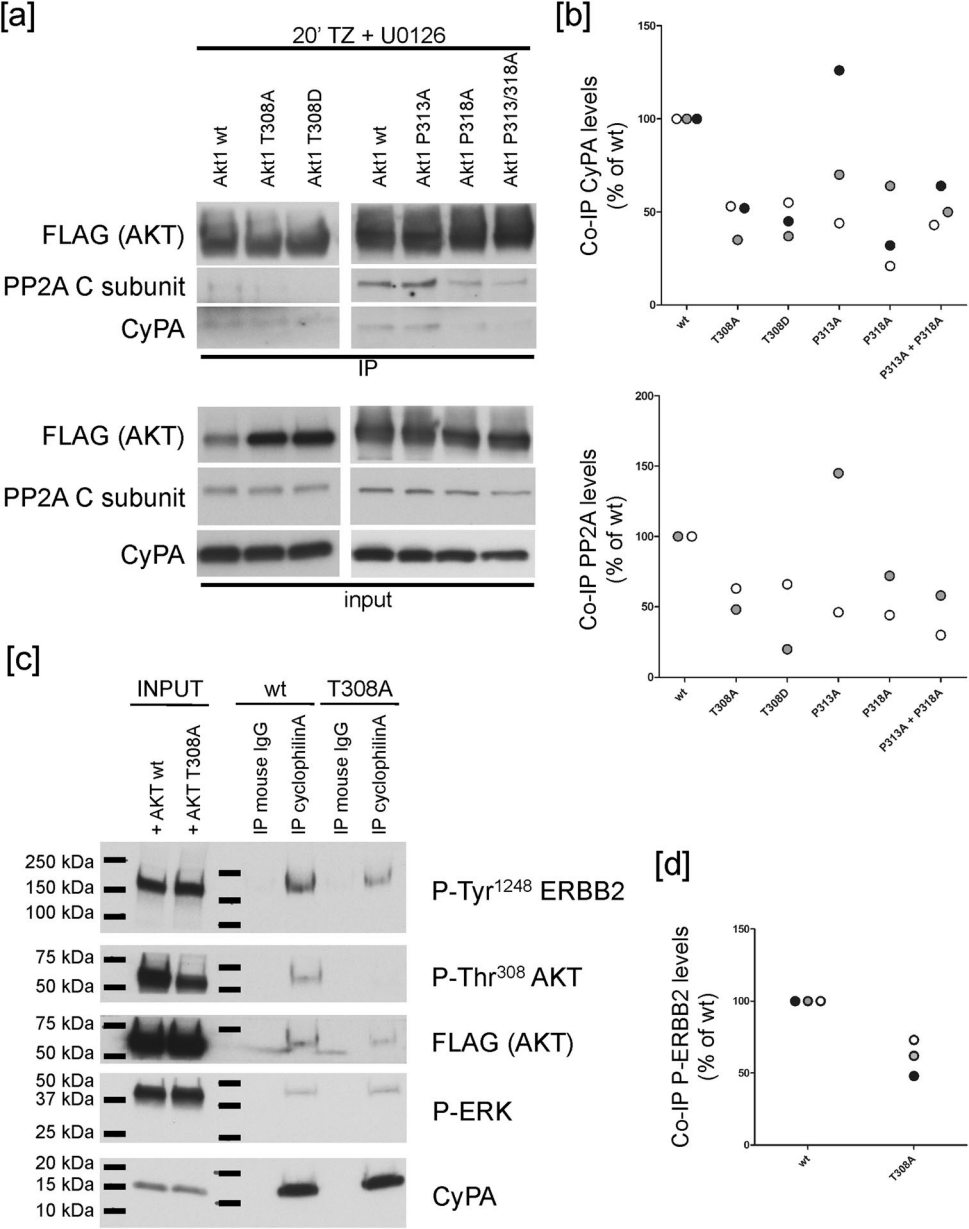
CyPA binds to AKT on Thr^308^ and Pro^318^ as PP2A. (**a**,**b**) SK-Br3 cells were transfected with constructs coding for FLAG-tagged AKT Thr or Pro mutants (as indicated) or wt AKT (as control). Cells were serum-starved for 20 min in the presence of 10 μM U0126, and incubated with 10 μg/ml TZ for 20 min. (**a**) Cells were immunoprecipitated with an antibody specific for the FLAG tag. (**b**) Amounts of co-immunoprecipitated (co-IP) proteins were normalized both on immunoprecipitated protein and their amount in the input. Individual data points obtained in two (PP2A) or three (CyPA) independent experiments are shown. T308A, p < 0.01; T308D, p < 0.01; P318A, p < 0.05; P313A + P318A, p = 0.01 Student's *t* test. (**c**,**d**) SK-Br3 cells were transfected with FLAG-tagged wt or T308A mutant AKT (as indicated). Cells were serum-starved and incubated with TZ for 20 min. Cells were immunoprecipitated with an antibody specific for CyPA. (**d**) CyPA immunoprecipitated approximately 40% less phospho-ERBB2 in the cells expressing mutant AKT, as compared to wt AKT (p < 0.05, Student's *t* test). The membrane was initially labelled with the anti-phospho AKT Ab, stripped and afterwards probed with the anti-FLAG Ab. The images of the full scan western blots are provided in Supplementary Fig. 11.

In order to identify the putative AKT moieties involved in CyPA binding, we generated FLAG-tagged wt and biomimetic mutants of the Thr^308^ residue of AKT1 (i.e. AKT^T308A^, not phosphorylatable, and AKT^T308D^, phosphomimetic) and performed IP experiments on cells expressing either one of the FLAG-AKT constructs. Unexpectedly, we found that upon 20 min of TZ/U0126 treatment, both biomimetic AKT mutants, with putative different outcomes on AKT activation, immunoprecipitated approximately 50% of CyPA and 60% of PP2A, as compared to wt AKT (Fig. 4a,b). These findings suggest that any substitution of Thr^308^ affects CyPA binding. Furthermore, as CyPA and PP2A do not directly interact (Fig. 3b,c), it is feasible that they may compete for the same binding site on AKT.

Since CyPA is a peptidyl-prolyl isomerase, we tested whether the proline residues of AKT close to Thr^308^ were relevant for the binding to CyPA. Thus, we mutagenized Pro^313^ and Pro^318^ of AKT to generate single and double mutants. We then treated cells expressing FLAG-tagged wt, AKT^P313A^, AKT^P318A^ or AKT^P313A/P318A^ constructs, with TZ/U0126 for 20 min, and performed IP experiments using an anti-FLAG antibody. Under these conditions, Pro^318^ substitution (either alone or in combination with P313A mutation) reduced the interaction between AKT and CyPA by about 60% (Fig. 4a,b). Similarly to Thr^308^, Pro^318^ substitution impaired also PP2A binding to AKT.

Thus, we can envisage that CyPA inhibits AKT de-phosphorylation, by competing with PP2A for the binding to AKT Thr^308^ residue. Co-IP experiments (Fig. 3) suggest that the recruitment of CyPA to the ERBB2 cytoplasmic tail could be necessary to displace CyPA from phospho-AKT, thus making the latter available to PP2A phosphatase activity.

Since CyPA is a cytosolic protein and it binds to phospho-AKT, which is phosphorylated on Thr^308^ by P-PDK1 on the PM, we hypothesized that CyPA is recruited onto ERBB2 upon Ab treatment by means of its interaction with phospho-AKT. Therefore, we immunoprecipitated CyPA in cells expressing FLAG-AKT^T308A^ treated with TZ for 20 min, and found that it binds approximately 40% less phospho-ERBB2 as compared to wt AKT (Fig. 4c,d). Thus, we conclude that TZ-induced ERK-dependent phospho-AKT recruitment to ERBB2 leads also to CyPA relocalization to the receptor.

### ERK and CyPA positively regulate ERBB2-Tyr^1248^ activation via a feedback loop involving ERBB2-Thr^701^ phosphorylation

Since the Ab-induced recruitment of the complex comprising ERK, CyPA and phospho-AKT onto ERBB2 depends on phospho-ERK, we reasoned that active ERK might either function as a scaffold protein, docking the complex onto the receptor cytoplasmic domain, or exert an enzymatic activity on ERBB2, rendering it accessible to the complex. Indeed, while both AKT and CyPA are not ERK substrates, as they do not comprise consensus sequences for ERK-mediated phosphorylation^42^, evidences from the literature suggest that ERBB2 can be a target of phospho-ERK^43^.

**Figure 5:**
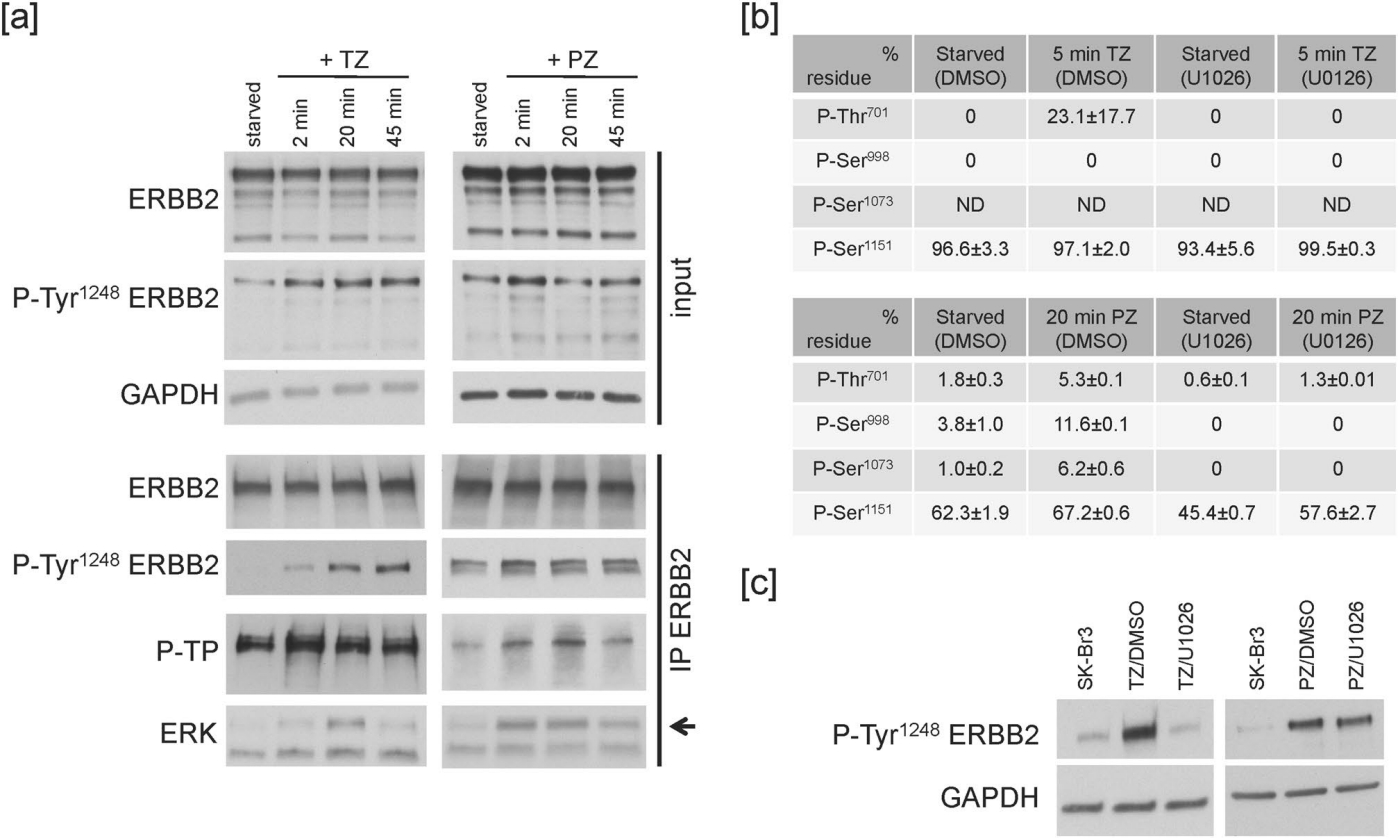
ERK controls a positive feedback keeping ERBB2 active. (**a**) Cells were serum-starved for 20 min, and treated with 10 μg/ml TZ or 20 μg/ml PZ at 37 °C for the indicated times. Lysates were immunoprecipitated with an ERBB2-specific antibody. Samples were analysed by WB. One experiment is shown as representative of two. (**b**) SK-Br3 cells were serum-starved for 20 min at 37 °C in the presence of U0126 or vehicle (DMSO), and treated with 10 μg/ml TZ for additional 5 min (upper table) or with 20 μg/ml PZ for additional 20 min (lower table) at 37 °C. Lysates were immunoprecipitated with an ERBB2-specific antibody. Samples were analysed by mass spectrometry. In the tables, mean ± st. dev. from one experiment is reported (out of two technical replicates). The results shown are representative of two independent biological replicates. (**c**) SK-Br3 cells were either left untreated or serum-starved for 20 min, in the presence of 10 μM U0126 or vehicle (DMSO), and treated with 10 μg/ml TZ or 20 μg/ml PZ at 37 °C for 20 min. Lysates were analysed by WB. For TZ, one experiment is shown as representative of nine; for PZ, one experiment is shown as representative of three. ERK inactivation reduces ERBB2 Tyr^1248^ phosphorylation induced by TZ or PZ down to 66% and 69% (where control sample is normalized as 100 percent), respectively (p = 0.02, paired Student's *t* test, for either TZ or PZ treatment). The images of the full scan western blots are provided in Supplementary Fig. 12.

In particular, ERBB2 intracellular domain comprises four target consensus sequences PX[S/T]P for ERK kinase^42^. To evaluate whether ERBB2 was a putative target for ERK activity upon Ab binding, we immunoprecipitated ERBB2 at different time-points following Abs treatment, and revealed ERK kinase consensus target sites with an antibody recognising anti-phospho-Threonine-Proline (P-TP) by WB^44^. The results showed that both TZ and PZ treatment induced a transient increase in P-TP signal, as compared to starved cells, with a peak around 2 min for TZ and 20 min for PZ (Fig. 5a). Noteworthy, under these conditions we observed that phospho-ERK co-immunoprecipitated with ERBB2 (Fig. 5a).

To identify the specific receptor residues specifically phosphorylated by ERK we immunoprecipitated ERBB2 after 5 min of TZ or TZ/U0126 and after 20 min of PZ or PZ/U0126 treatment and performed label-free quantitative mass spectrometry. Peptide analysis revealed that Thr^701^ was specifically phosphorylated upon both TZ (Fig. 5b, upper table) and PZ (Fig. 5b, lower table) treatments, but not in the corresponding Ab/U0126 conditions. Noteworthy, PZ treatment induced the ERK-dependent phosphorylation of additional sites, namely Ser^998^ and Ser^1073^ (Fig. 5b, lower table).

As it has been reported that the ERK-mediated phosphorylation of a Thr residue in the juxtamembrane region of the EGFR cytoplasmic domain modulates the EGF-induced receptor downstream signaling^44,45^, we tested whether ERK would control the activation of ERBB2 as well. To this end, we assessed the phosphorylation state of the ERBB2 Tyr^1248^ residue. Tyr^1248^ is an autophosphorylation site^46^ in the receptor C-terminal tail and its phosphorylation is considered a pre-requisite for ERBB2 signaling, and transforming activity^2^. By WB, we observed that both TZ and PZ treatments induced a strong Tyr^1248^ phosphorylation in SK-Br3 cells (Fig. 5a,c), and that this response was lowered in the corresponding Ab/U0126 condition (Fig. 5c). These data suggest that the Ab-induced ERK-dependent phosphorylation of ERBB2 juxtamembrane region (Thr^701^) may function as a positive feedback mechanism to promote ERBB2 C-terminal (Tyr^1248^) activation, and downstream signaling.

**Figure 6:**
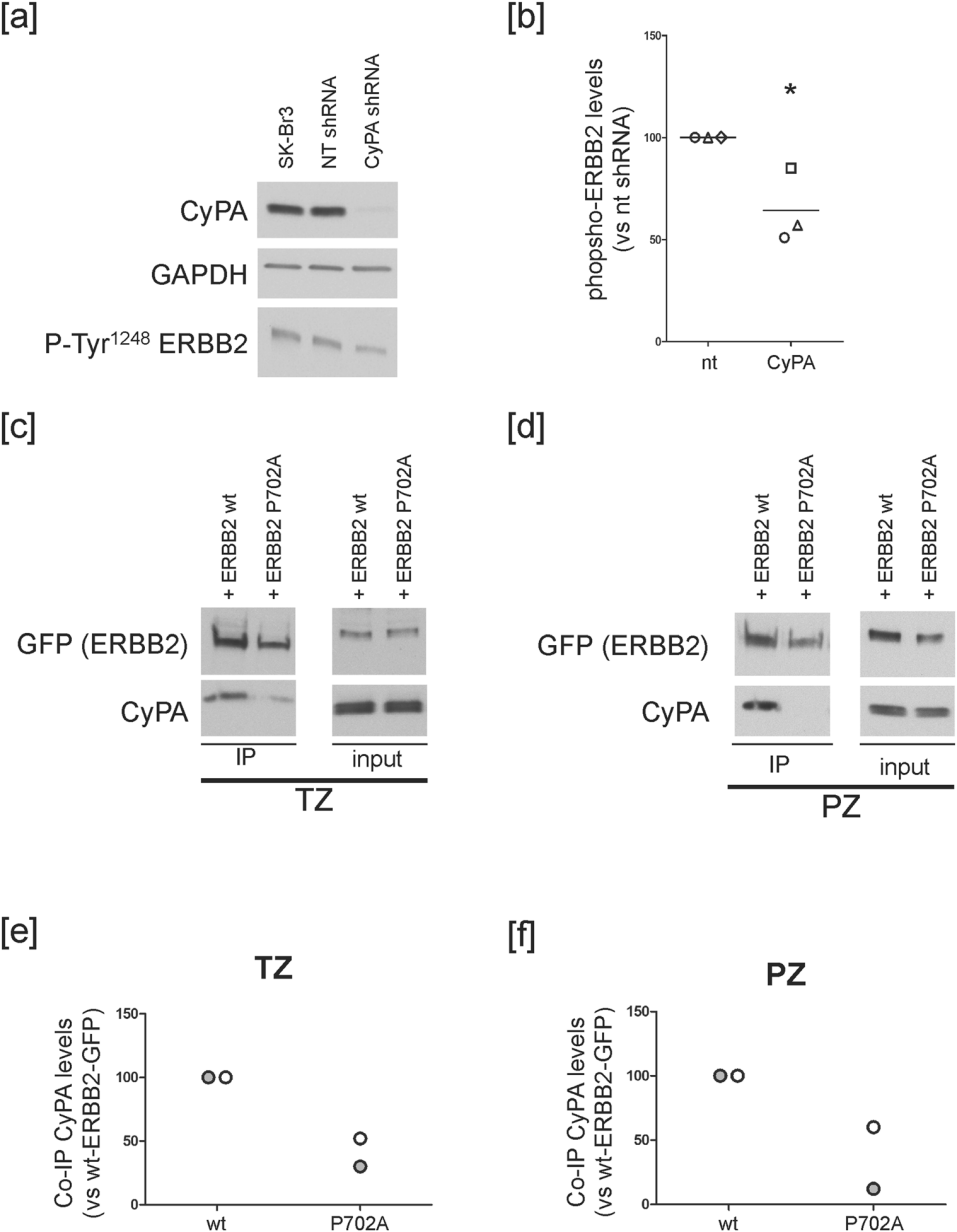
CyPA partake to a positive feedback keeping ERBB2 active. (**a**,**b**) SK-Br3 cells were transduced with a non-targeting (NT) or a CyPA-specific shRNA for 15 days. (**a**) Lysates were analysed by WB. One experiment is shown as representative of three. (**b**) Graph shows phospho-ERBB2 levels (p < 0.05, Student's *t* test). Lines indicate mean out of three independent experiments. Data points are shape-coded. (**c**–**f**) SK-Br3 cells were transfected with constructs coding for GFP-tagged P702A ERBB2 mutant or wt ERBB2 (as control). Cells were serum-starved for 20 min, and incubated with 10 μg/ml TZ (**c**) or 20 μg/ml PZ (**d**) at 37 °C for 20 min. Lysates were immunoprecipitated with an antibody specific for the GFP tag. (**c**,**d**) Samples were analysed by WB. (**e**,**f**) Amounts of co-immunoprecipitated (co-IP) CyPA were normalized both on immunoprecipitated protein (GFP) and their amount in the input. Individual data points obtained in two independent experiments are shown. The images of the full scan western blots are provided in Supplementary Fig. 13.

Since ERK ability to phosphorylate its substrates has been shown to be sensitive to the conformation of the substrate backbone [(Ser/Thr)-Pro]^47^, in the ERBB2 context, we hypothesized that CyPA could be involved in the positive feedback loop controlling ERBB2 C-terminal tail phosphorylation. To test this hypothesis, we analyzed the levels of ERBB2 Tyr^1248^ phosphorylation in SK-Br3 cells silenced for CyPA, and found significantly reduced levels of phospho-ERBB2 [50% as compared to control nt shRNA-transduced cells, Fig. 6a,b], supporting the conclusion that CyPA positively modulates ERBB2 activation state.

Thus, as we have shown that the immunophilin recruitment onto ERBB2 depends on phospho-ERK (Fig. 3), we mutagenized the Pro^702^ residue of the receptor into Ala, expressed the recombinant mutant GFP-tagged proteins in SK-Br3 cells, and treated them with TZ or PZ. We found that ERBB2^P702A^-GFP recruits less CyPA as compared to wt ERBB2-GFP upon treatment with either Ab (Fig. 6c–f).

Altogether, these results suggest that upon Ab treatment: (i) ERK takes part to a positive feedback loop keeping ERBB2 active through the phosphorylation of ERBB2 Thr^701^ residue; (ii) CyPA participate to the regulation of ERBB2 activation and (iii) Pro^702^ represents the ERBB2 binding site for CyPA.

## Discussion

While almost undetectable or expressed at very low levels in normal tissues^1^, ERBB2 is overexpressed in several carcinomas, including breast cancers that display a more aggressive clinical course and a worst outcome compared to non ERBB2-positive BrCa^4,5^. ERBB2 represents the preferred partner of the other ERBB receptors^14^, and ERBB2-containing heterodimers are more oncogenic than other ERBB combinations^15–19^. Thus, it is widely accepted that ERBB2 has a pro-oncogenic role. However, its precise function and downstream signaling pathway have not been assessed: the absence of a known ligand for ERBB2 has been the major difficulty to address this issue, and most studies have relied on the stimulation of the partner receptor in ERBB2-containing heterodimers.

Here, we show that, at variance to ligand activation of ERBB2-ERBB3 heterodimers by HRG or of EGFR-ERBB2 heterodimers by EGF, triggering both ERK and AKT signaling pathways, treatment with ERBB2-targeting Abs leads mainly to receptor homodimerization and to an ERK-dependent AKT inactivation. This signaling is ERBB2-specific and appears not to depend on ERBB2 heterodimers as in the context of the breast cancer cell line used here (expressing negligible ERBB4 levels), silencing of EGFR and ERBB3 does not impair the ERK-dependent AKT de-phosphorylation elicited by Abs. On the other hand, the very early transient peak in AKT phosphorylation, preceding its de-phosphorylation, is likely due to ERBB2 activity in a heterodimeric context. Indeed, at variance to EGFR and ERBB3, ERBB2 has no binding sites for PI3K in its cytosolic domain^7^, thus it cannot directly stimulate AKT phosphorylation. Hence, Ab binding to ERBB2 leads to two independent events: in the heterodimeric context, ERBB2 cooperates on the phosphorylation of AKT induced by the partner; in the homodimeric form, the receptor triggers the specific negative modulation of the AKT pathway, thus counteracting the pro-survival activity promoted by ERBB heterodimers.

This agonist role played by the Abs on ERBB2 is further supported by structural studies/simulations showing that TZ or PZ binding to ERBB2 promotes conformational changes of the receptor, similar to those reported for EGFR upon binding to EGF (reviewed in^48^), in particular: (1) fluctuation in domain II of the receptor upon TZ binding and in domain IV upon PZ binding^49^, (2) displacement of the transmembrane domain, which loses its interaction with the inner leaflet of the membrane bilayer, upon TZ binding^50^ and (3) interference with the antiparallel alignment of the juxtamembrane domains upon TZ binding^50^.

The Ab-induced ERK-dependent signaling cascade triggering AKT de-phosphorylation involves both PP2A Ser/Thr phosphatases and the cytosolic immunophilin CyPA. Immunophilins exert different important functions in the cell, acting either as molecular chaperones assisting and/or correcting protein folding, or as enzymes catalysing the *cis–trans* isomerization at Pro residues^51^. It has been reported that the immunophilin Pin1 is overexpressed in breast cancer and that its up-regulation is prevalent in ERBB2-positive tumours^52,53^; however, its silencing or functional inhibition does not potentiate TZ activity, possibly because it accelerates receptor degradation^54^. Similarly to Pin1, CyPA is found overexpressed in different types of tumours, including breast cancer, and in some reports a correlation between CyPA overexpression and malignant transformation has been reported^55,56^.

Our knock down experiments show that CyPA counteracts Ser/Thr phosphatase activity on phospho-AKT, possibly by competing with PP2A for binding to phospho-AKT, as suggested by IP experiments with AKT mutants. Upon Ab treatment, a complex comprising phospho-ERK, CyPA and phospho-AKT is recruited onto the cytoplasmic domain of ERBB2, and a reduction in phospho-AKT levels is observed. Altogether, these findings support the hypothesis that the recruitment of CyPA onto ERBB2, induced by Ab treatment, renders phospho-AKT accessible to Ser/Thr phosphatase activity, explaining the results we obtained with the phosphatase inhibitors, i.e. that TZ regulates the association of the phosphatase to its substrate rather than the catalytic activity. This unanticipated role of Ser/Thr phosphatases in TZ action reconciles published observations implicating these enzymes both in breast carcinogenesis^24^ and in the onset/development of resistance to TZ treatment^57^. Furthermore, our findings are in agreement with published data showing that AKT Ser^473^ de-phosphorylation is essentially mediated by PP2C phosphatases, whereas AKT Thr^308^ de-phosphorylation is performed by PP2A holoenzymes^36,37^.

**Figure 7:**
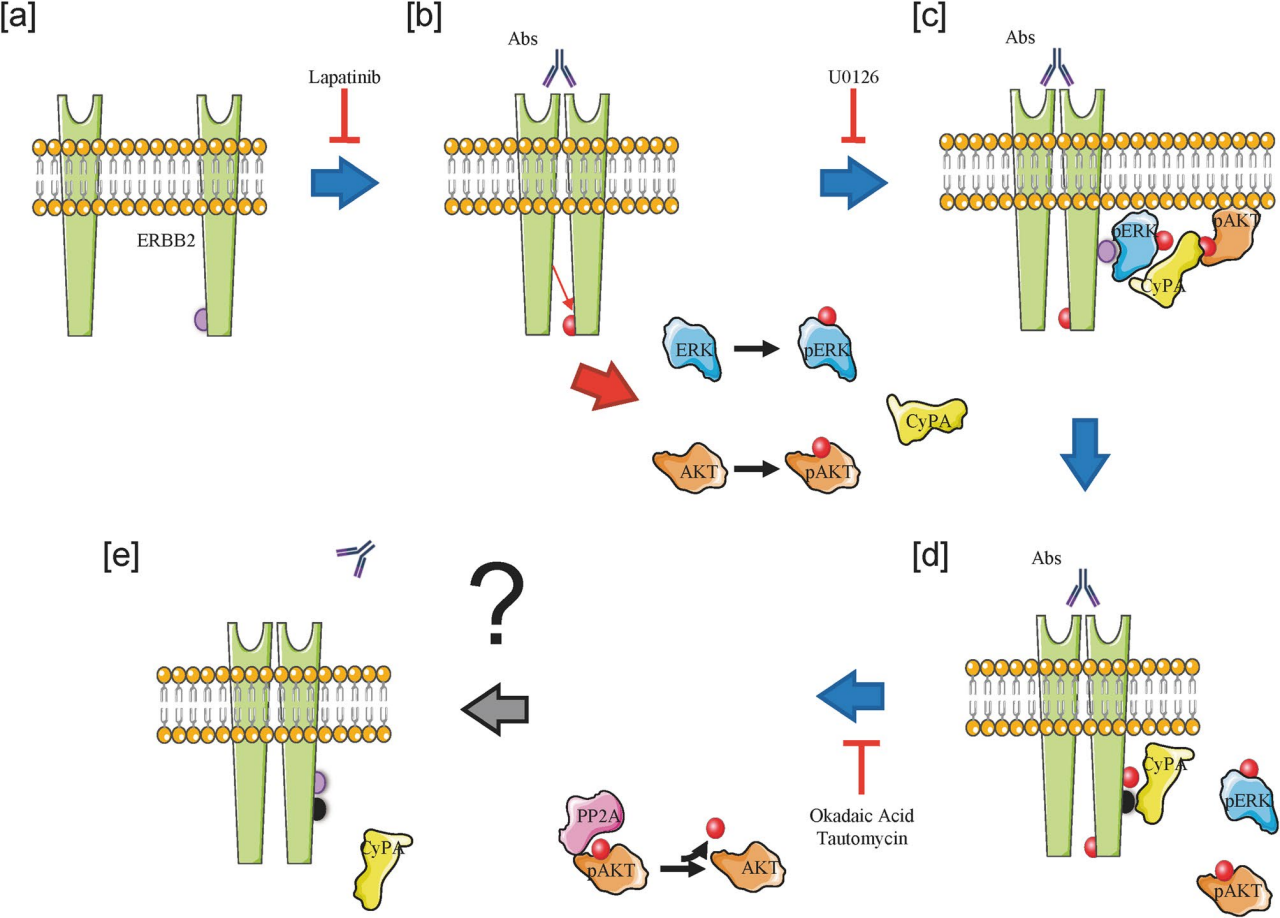
Proposed ERBB2 intracellular mechanism of action. (**a**,**b**) Abs treatment induces ERBB2 homodimerization and transactivation of Tyr^1248^. (**b**,**c**) ERBB2 C-terminal domain phosphorylation induces MAPK (ERK) pathway activation. (**c**,**d**) Active ERK binds to and phosphorylates ERBB2 Thr^701^ residue, sustaining ERBB2 C-terminal domain phosphorylation (positive feedback loop), and fostering the recruitment of phospho-AKT and CyPA onto the receptor. (**d**,**e**) CyPA binding to ERBB2 Pro^702^ residue possibly sequesters the immunophilin from the interaction with phospho-AKT, which becomes accessible to and is de-phosphorylated by PP2A Ser/Thr phosphatase.

ERK activity is pivotal in Ab-mediated ERBB2 signaling, as it plays a dual role: it promotes the recruitment of the ERK/CyPA/phospho-AKT complex onto ERBB2, leading to AKT de-phosphorylation, and the full activation of the receptor C-terminal domain, through the phosphorylation of the Thr^701^ residue in the juxtamembrane domain, identified by mass spectrometry (see model in Fig. 7).

The role of phospho-ERK in the full activation of ERBB2 by Abs is highlighted by the observation that the use of MEK inhibitors reduces the phosphorylation levels of the ERBB2 Tyr^1248^ residue, which is required for ERBB2 signaling, and its transforming activity^2,46^. Noteworthy, this positive feedback functions at odds with what has been previously reported. In particular, EGF has been shown to promote an ERK-dependent phosphorylation of EGFR Thr^693^, homologous to ERBB2 Thr^701^, which negatively regulates the Tyr phosphorylation of the receptor C-terminal domain^44,45,58^. Similarly, 12-*O*-Tetradecanoylphorbol-13-acetate (TPA)-induced ERK-dependent ERBB2 Thr^701^ phosphorylation has been shown to abrogate ERBB2 phosphorylation on Tyr^1248^ and Tyr^1196^^43^. Noteworthy, EGF binding to EGFR activates both the MAPK and the AKT signaling pathways, whereas ERBB2-targeted Abs have opposite effects on the two cascades, as the direct activation of ERK is propaedeutic to AKT inactivation. By contrast, TPA has been reported to stimulate ERK and inhibit AKT activity, by interfering with the heterodimerization between ERBB2 and ERBB3^43^. On the contrary, our results show that Abs function mainly on ERBB2 homodimers, thus providing a possible explanation for the discrepancies with the published observation.

Furthermore, phospho-ERK renders ERBB2 accessible to CyPA and phospho-AKT upon Ab treatment. Here, we provide evidence for the ERK-dependent recruitment of CyPA to the membrane-localized ERBB2, by both biochemical (IP) and morphological (dSTORM super-resolution microscopy) approaches, suggesting that CyPA is a key molecular switch in ERBB2 downstream signaling. Ab-bound ERBB2 is phosphorylated by ERK and the kinase ability to phosphorylate its substrates has been shown to be sensitive to the conformation of the substrate backbone [(Ser/Thr)-Pro]^47^. Furthermore, a peptidyl-prolyl isomerase has been shown to target and isomerize the Pro residue next to the Ser/Thr residue phosphorylated by ERK^59^. Indeed, our results show that CyPA silencing significantly reduces the levels of phospho-ERBB2, thus supporting the involvement of CyPA in the regulation of ERBB2 phosphorylation and activation. Moreover, we identified ERBB2 Pro^702^ as the binding site for CyPA (see model in Fig. 7). However, whether the isomerase activity of CyPA is involved in ERK-mediated receptor phosphorylation still remains an open issue.

In conclusion, we identified and characterized for the first time an ERBB2-specific downstream anti-oncogenic signaling induced by treatment with Ab, negatively modulating the AKT pro-survival pathway, and recognized CyPA as a key regulator of this process.

These data shed new light on a dichotomous role of ERBB2: pro-oncogenic in the heterodimeric context and anti-proliferative in the homodimeric form. This previously unrecognized role of ERBB2 as a negative modulator of other ERBBs activity opens new perspectives for the treatment of ERBB2-positive carcinomas.

## Methods

### dSTORM super-resolution microscopy and pair cross-correlation

dSTORM super-resolution imaging was performed on a Leica SR GSD 3D TIRF microscope equipped with 300 mW 532 nm and 642 nm lasers; a 30 mW 405 nm laser; a Leica HCX PL APO 160 ×/1.43 Oil CORR GSD objective; a quad-band filter set with 417 nm, 496 nm, 544 nm, 655 nm dichroic and 421–477 nm, 497–519 nm, 547–621 nm, 666–732 nm emission bands. Images were collected with an Andor iXon Ultra 897 EM-CCD camera over an area of 18 × 18 µm. For TZ, samples were immunostained for CyPA (Alexa 647) and ERBB2 (Alexa 568) as described above. During acquisition, samples were embedded in MEA-Glucose Oxidase imaging buffer [PBS, 10 mM β-mercaptoethylamine (MEA) pH 7.4, 10% glucose (w/v), 0.5 mg/ml glucose oxidase, 40 µg/ml catalase] freshly prepared before each acquisition. In order to minimize photobleaching, Alexa 647 was imaged first, followed by Alexa 568. Fluorophores were initially pumped into dark states by illumination with 642 nm or 532 nm lights at 80% laser power until single fluorophore blinking was observed (< 15 s). Subsequently, single molecules were imaged at 60% laser power with a frame rate of 140 frames per second until a total of 10^6^ (CyPA) and 10^5^ (ERBB2) events was reached, using a detection threshold of 20 photons and 40 photons for event recognition, respectively. For PZ, samples were immunostained for CyPA (Alexa 647) and ERBB2 (Alexa 555) as described previously. During acquisition, samples were embedded in dSTORM Super Resolution buffer (Abbelight). In order to minimize photobleaching, Alexa 647 was imaged first, followed by Alexa 555. Fluorophores were initially pumped into dark states by illumination with 642 nm or 532 nm lights at 40% and 60% laser power until single fluorophore blinking was observed (< 15 s), respectively. Subsequently, single molecules were imaged at 40% and 60% laser power with a frame rate of 140 frames per second until a total of 10^6^ (CyPA) and 2 × 10^5^ (ERBB2) events was reached, using a detection threshold of 40 photons and 60 photons for event recognition, respectively. The final images were calculated using a pixel size of 20 nm in the histogram mode.

CyPA recruitment on ERBB2 was assessed by performing pair cross-correlation analysis on 1 × 1 µm regions of interest (ROIs), as previously described^41^.

### Fab fragment preparation

TZ and PZ were enzymatically cleaved on immobilized papain for 10 h at 37 °C, according to Thermo Scientific Pierce Fab Preparation Kit instructions. Digestion was verified by SDS-page, and Fab fragments purified using high recovery regenerated cellulose membranes (Amicon Ultra centrifugal filter units) with 100 kDa (to remove uncleaved whole Ab) and 30 kDa (to remove the Fc portion) cut-offs. Fab concentration was assessed by Bradford assay (Bio-Rad).

### Immunoprecipitation (IP)

#### p-Thr308 AKT and CyPA interactors identification

SK-Br3 cells were either left untreated, or serum-starved for 20 min at 37 °C either in the absence or in the presence of U0126 and treated with TZ for additional 20 min at 37 °C. Before cell lysis, cells were washed twice with r.t. PBS and treated with 1 mM DSP in PBS for 10 min at r.t. The cross-linker was quenched by incubation with 20 mM Tris for 5 min at r.t. Cells were washed twice with cold PBS, scraped in lysis buffer (containing 20 mM Hepes, 150 mM NaCl, 1% Triton-X100, 10% glycerol, and protease inhibitor cocktail, phosphatase inhibitor cocktail, 1 mM NaF, 1 mM sodium orthovanadate, 1 mM beta-glycerophosphate), incubated for 10 min on ice and sonicated. Cells were incubated for 45 min on ice, and centrifuged at 16,000*g* for 5 min at 4 °C to remove nuclei and membranes. Protein concentrations were determined (BCA protein assay). Meanwhile, antibody binding to protein G dynabeads was performed with the protocol described in^60^ with slight modifications. The antibody-crosslinked beads (50 μl) were incubated overnight at 4 °C with lysates of SK-Br3 cells. The following day, after removing the supernatant (unbound) and washing with buffer (containing 20 mM Hepes, 300 mM NaCl, 1% Triton-X100, 10% glycerol, and protease inhibitor cocktail, phosphatase inhibitor cocktail, 1 mM NaF, 1 mM sodium orthovanadate, 1 mM beta-glycerophosphate) for 5 × 5 min, the immunoprecipitates were eluted with 30 μl of 2 × Laemmli buffer without reducing agent at 37 °C for 10 min. The eluate was transferred into a new tube, and samples were subjected to SDS-PAGE separation for Coomassie and mass spectrometry analysis. Alternatively, the eluate was transferred into a new tube, 10% β-mercaptoethanol added to cleave DSP, and samples were subjected to SDS-PAGE separation for Western blotting.

#### AKT mutants analysis

SK-Br3 cells were transfected with constructs coding for FLAG-tagged AKT mutants, on the following day serum-starved for 20 min at 37 °C in the presence of U0126, and treated with TZ for additional 20 min at 37 °C. Before cell lysis, cells were washed twice with PBS and cross-linked with DSP, as described above. The cross-linker was quenched by incubation with 20 mM Tris for 5 min at r.t. Cells were washed with PBS, scraped in lysis buffer, incubated for 10 min on ice and sonicated. After 45 min on ice, cells were centrifuged at 16,000*g* for 5 min at 4 °C. Protein concentrations were quantified by BCA. Afterwards, anti-FLAG magnetic beads were incubated with 4–5 mg of protein lysates of transfected SK-Br3 cells for 3 h at 4 °C. After removing the supernatant (unbound) and washing with washing buffer (see above) for 5 × 5 min, the immunoprecipitates were eluted with 40 μl/sample of 2 × Laemmli buffer at 95 °C for 10 min, and then underwent SDS-PAGE separation for Western blotting.

#### ERBB2 phospho-peptide analysis

SK-Br3 cells were serum-starved for 20 min at 37 °C either in the absence or in the presence of U0126 and treated with TZ for additional 5 min at 37 °C or with PZ for additional 20 min at 37 °C. Cells lysates were prepared and their protein concentration determined as described above. Meanwhile, protein G dynabeads were incubated with antibody for 30 min at r.t. The antibody-bound beads (900 μl/sample) were incubated overnight at 4 °C with 15–20 mg of protein lysates of SK-Br3 cells. The following day, the supernatant (unbound) was removed, the beads were washed with washing buffer (see above) 5 times for 5 min, and the immunoprecipitates were eluted with 300 μl/sample of 2 × Laemmli buffer at 95 °C for 10 min (450 rpm agitation). The eluates were transferred into a new tube, and the samples were analysed by SDS-PAGE separation on 6% gels followed by either Coomassie staining and mass spectrometry or Western blotting.

#### ERBB2 dimerization analysis

SK-Br3 cells were serum-starved for 20 min at 37 °C and treated with TZ/PZ or Fab for additional 10 min at 37 °C. Before cell lysis, cells were washed with PBS and treated with 1 mM DTSSP in PBS for 10 min at r.t. The cross-linker was quenched as described above. Lysates were prepared as described above. Meanwhile, protein A dynabeads were incubated with rabbit anti-human antibody for 30 min at r.t. (to immunoprecipitate only the ERBB2 cohort bound to the treatment, TZ/PZ or Fab). One third of protein A dynabeads (already coated with rabbit anti-human antibody) were incubated with TZ/PZ for 30 min at r.t. (to immunoprecipitate the starved sample). The antibody-bound beads (450 μl/sample) were incubated overnight at 4 °C with 7.5 mg of protein lysates of SK-Br3 cells. The following day, after removing the supernatant (unbound) and washing with washing buffer (see above) for 5 × 5 min, the immunoprecipitates were eluted with 200 μl/sample of 2 × Laemmli buffer without reducing agent at 95 °C for 10 min (450 rpm agitation). The eluates were transferred into a new tube, and the samples underwent SDS-PAGE separation on 6% gels for Coomassie and mass spectrometry analysis or Western blotting.

#### ERBB2 mutants analysis

SK-Br3 cells were transfected with constructs coding for GFP-tagged ERBB2 mutants, on the following day serum-starved for 20 min at 37 °C, and treated with TZ or PZ for additional 20 min at 37 °C. Cells were washed with r.t. PBS and treated with DSP as described above. The cross-linker was quenched by incubation with 20 mM Tris for 5 min at r.t. Cell lysates were prepared as described above. Meanwhile, anti-rabbit IgG F(ab')2 Fragment-conjugated Sepharose beads were incubated with anti-GFP antibody for 90 min at r.t. (to immunoprecipitate only the ERBB2 cohort comprising at least one mutated ERBB2 partner). The antibody-bound beads (50 μl/sample) were incubated overnight at 4 °C with 5–6 mg of protein lysates of transfected SK-Br3 cells. The following day, the supernatant (unbound) was removed, the beads were washed with washing buffer (see above) for 5 × 5 min, and the immunoprecipitates were eluted with 50 μl/sample of Laemmli buffer at 95 °C for 10 min (500 rpm agitation). The eluates were subjected to SDS-PAGE separation and WB.

### Antibody crosslinking to dynabeads

Antibody binding to dynabeads was performed following the protocol reported in^60^ with slight modifications. In particular, protein G dynabeads slurry (100 μl) was washed twice with 400 μl PBS buffer, and incubated with 18 μg phospho-AKT or 20 μg CyPA specific antibody (or rabbit/mouse IgG as control, respectively) prepared in PBS (200 μl final volume) at r.t. for 30 min on a wheel. The supernatant was discarded and the beads were washed three times with 600 μl PBS, followed by incubation with 200 μl DSS solution (20 μl 10 × PBS + 160 μl H_2_O + 20 μl 100 mM DSS in DMSO) at r.t. for 60 min on a wheel. After removing the supernatant the beads were washed three times with 200 μl 1 M Tris–HCl pH 7.4, twice with 600 μl PBS buffer containing 1% Tween-20, then once with 600 μl PBS. The antibody-crosslinked beads (50 μl) were incubated overnight at 4 °C with 4 mg or 1.5 mg (for phospho-AKT or CyPA, respectively) of lysate of SK-Br3 cells.

### Mass spectrometry analysis

#### p-Thr308 AKT interactors identification

The entire lanes were individually cut in nine bands and in-gel digested prior to mass spectrometry analysis. In particular, the bands, once excised from the gels, were de-stained, sequentially reduced, alkylated and digested overnight with sequencing-grade trypsin, as previously described^61^. Aliquots of the sample containing tryptic peptides were desalted using StageTip C18 (Thermo Scientific) and analysed by nLC-MS/MS using an LTQ-Orbitrap (Thermo Scientific, Bremen, Germany) equipped with a nano-electrospray ion source (Proxeon Biosystems) and an nHPLC Easy LC (Proxeon Biosystems). Peptide separations occurred on a homemade (75 µm i.d., 25 cm long) reverse phase silica capillary column, packed with 3-µm ReproSil-Pur 120 C18-AQ (Dr. Maisch GmbH, Germany). The nLC-MS/MS was performed with the protocol described in^62^ with slight modifications. A gradient of eluents A (distilled water with 2% v/v acetonitrile and 0.5% v/v acetic acid) and B (acetonitrile and 20% v/v distilled water with 0.5% v/v acetic acid) was used to achieve separation (150 nl/min flow rate), from 8% B to 50% B in 50 min. Full scan spectra were acquired with the lock-mass option, resolution set to 60,000 and mass range from *m*/*z* 300 to 1750 Da. The ten most intense doubly and triply charged ions were selected and fragmented in the ion trap. All MS/MS samples were analysed using Mascot (version 2.6, Matrix Science) search engine to search the UniProt_Human Complete Proteome_cp_hum_20170315 (92,919 sequences; 36,868,442 residues). Searches were performed with 3-missed cleavages allowed, carbamidomethylation on cysteine as fixed modification, protein N-terminus-acetylation, methionine oxidation and addition of 145.0198 Da on lysine and protein N-terminus (CAMthiopropanoyl modification) due to the cross-linking as variable modifications. Mass tolerance was set to 5 ppm and 0.6 Da for precursor and fragment ions, respectively.

#### ERBB2 post translational modifications (PTM) assessment

The band, corresponding to ERBB2 protein, was excised from the gel, de-stained, sequentially reduced, alkylated with iodoacetamide and digested overnight with sequencing-grade trypsin, as above described. The peptides were extracted from the bands and subdigested overnight with endoproteinase Glu-C (Roche Diagnostics). The phosphopeptides were enriched on TiO_2_ resin and subsequently desalted using POROS Oligo R3 reversed-phase resin, as previously reported^63^. The flow-through peptides were also desalted using a Stage Tip C18 (Thermo Scientific). All the peptide mixtures were analysed by nLC-MS/MS using a Q-Exactive mass spectrometer (Thermo Scientific, Bremen, Germany) equipped with a nano-electrospray ion source (Proxeon Biosystems) and a nUPLC Easy-nLC 1000 (Proxeon Biosystems) as described in^64^ with slight modifications. Peptide separations occurred on a homemade (75 µm i.d., 12 cm long) reverse phase silica capillary column, packed with 1.9-µm ReproSil-Pur 120 C18-AQ (Dr. Maisch GmbH, Germany). A gradient of eluents A (distilled water with 0.1% v/v formic acid) and B (acetonitrile with 0.1% v/v formic acid) was used to achieve separation (300 nl/min flow rate), from 0% B to 45% B in 45 min. Full scan spectra were acquired with the lock-mass option, resolution set to 70,000 and mass range from *m*/*z* 300 to 2000 Da. The ten most intense doubly and triply charged ions were selected and fragmented in the ion trap^64^. The experiments were performed in technical duplicates and biological duplicates. In order to quantify the amount of phosphorylation on each site, the raw data were loaded into the MaxQuant software version 1.5.2.8. Searches were performed against the UniProt_Human Complete Proteome_cp_hum_20170315 (92,919 sequences; 36,868,442 residues), with trypsin + Glu-C as proteolytic enzymes, 2-missed cleavages allowed, carbamidomethylation on cysteine as fixed modification, protein N-terminus-acetylation, methionine oxidation and phosphorylation on Ser/Thr/Tyr as variable modifications. Mass tolerance was set to 5 ppm and 20 ppm for precursor and fragment ions, respectively. The intensities of precursors were used for the label-free protein quantification. Peptides and proteins were accepted with a FDR less than 1%, two minimum peptides per protein with one unique.

#### ERBB2 dimerization assessment

The high molecular weight bands containing ERBB2 protein, as indicated by WB analysis, were excised from the gel, de-stained, sequentially reduced, alkylated with iodoacetamide and digested overnight with sequencing-grade trypsin, as previously described^61,65^. The peptides were extracted from the bands, desalted using a Stage Tip C18 (Thermo Scientific) and analysed by nLC-MS/MS using a Q-Exactive mass spectrometer (Thermo Scientific, Bremen, Germany) equipped with a nano-electrospray ion source (Proxeon Biosystems) and an UPLC Easy-nLC 1000 (Proxeon Biosystems), as above reported. The experiments were performed in technical triplicates and biological duplicates. In order to quantify the amount of proteins in the high-molecular weight bands, the raw data were loaded into the MaxQuant software version 1.5.2.8. Searches were performed against the UniProt_Human Complete Proteome_cp_hum_20180228 (93,786 sequences; 37,179,059 residues), with the same parameters reported above plus the addition of 145.0198 Da on lysine and protein N-terminus (CAMthiopropanoyl modification) due to the cross-linking as variable modifications. Mass tolerance was set to 5 ppm and 20 ppm for precursor and fragment ions, respectively. The intensities of precursors were used for the label-free protein quantification. Peptides and proteins were accepted with an FDR less than 1%, two minimum peptides per protein with one unique. In order to normalize the amount of proteins, the rabbit anti-human antibody, which was equally added to the samples during the IP, was also quantified. In this case, when using the MaxQuant software for the label free analysis of the bands corresponding to the Ab (Supplementary Fig. 4) searches were performed against the UniProt_Rabbit Complete Proteome_cp_rabbit_20180228. All the other parameters were as above reported. To quantify the relative abundance of heterodimers vs homodimers, we normalized the intensities of EGFR (to estimate heterodimers) and ERBB2 (to estimate homodimers) peptides (per μg of immunoprecipitate) for the equivalent peptides obtained per μg of control SK-Br3 lysate.

### Ethics statement

All experimental protocols were approved by the Institutional Ethical Board of the European Institute of Oncology (IEO, Milan, Italy); all patients recruited for this study signed informed consent under these ethics, and all methods were carried out in accordance with relevant guidelines and regulations.

## Supplementary information

Supplementary Information.

Supplementary Table S1.

Supplementary Table S2.
