# Author Correction: Direct stimulation of ERBB2 highlights a novel cytostatic signaling pathway driven by the receptor Thr^701^ phosphorylation

**DOI:** 10.1038/s41598-021-97699-1

**Published:** 2021-09-08

**Authors:** Marco Gaviraghi, Andrea Rabellino, Annapaola Andolfo, Matthias Brand, Chiara Brombin, Paola Bagnato, Giuseppina De Feudis, Andrea Raimondi, Alberta Locatelli, Daniela Tosoni, Davide Mazza, Luca Gianni, Giovanni Tonon, Yosef Yarden, Carlo Tacchetti, Tiziana Daniele

**Affiliations:** 1 Division of Experimental Oncology, Istituto di Ricovero e Cura a Carattere Scientifico (IRCCS) San Raffaele Scientific Institute, via Olgettina 60, 20132 Milan, Italy; 2 Department of Experimental Medicine, University of Genoa, via De Toni 14, 16132 Genoa, Italy; 3 Protein Microsequencing Facility, Istituto di Ricovero e Cura a Carattere Scientifico (IRCCS) San Raffaele Scientific Institute, via Olgettina 60, 20132 Milan, Italy; 4 Experimental Imaging Centre, Istituto di Ricovero e Cura a Carattere Scientifico (IRCCS) San Raffaele Scientific Institute, via Olgettina 58, 20132 Milan, Italy; 5 University Centre for Statistics in the Biomedical Sciences, Vita-Salute San Raffaele University, via Olgettina 58, 20132 Milan, Italy; 6 Department of Oncology, Istituto di Ricovero e Cura a Carattere Scientifico (IRCCS) San Raffaele Scientific Institute, via Olgettina 60, 20132 Milan, Italy; 7 Department of Experimental Oncology, IEO, European Institute of Oncology IRCCS, 20100 Milan, Italy; 8 Center for Translational Genomics and Bioinformatics, Istituto di Ricovero e Cura a Carattere Scientifico (IRCCS) San Raffaele Scientific Institute, via Olgettina 60, 20132 Milan, Italy; 9 Weizmann Institute of Science, 76100 Rehovot, Israel; 10 Vita-Salute San Raffaele University, via Olgettina 58, 20132 Milan, Italy; 11 Present Address: QIMR Berghofer Medical Research Institute, Brisbane, QLD 4029 Australia; 12 Present Address: CeMM Research Center for Molecular Medicine of the Austrian Academy of Sciences, 1090 Vienna, Austria

Correction to: *Scientific Reports* 10.1038/s41598-020-73835-1, published online 09 October 2020

The original version of this Article contained an error in Affiliation 7, which was incorrectly given as ‘European Institute of Oncology, 20100, Milan, Italy’. The correct affiliation is listed below:

Department of Experimental Oncology, IEO, European Institute of Oncology IRCCS, 20100, Milan, Italy.

The original Article has been corrected.

