# Supplementary material for: Direct stimulation of ERBB2 highlights a novel cytostatic signaling pathway driven by the receptor Thr701 phosphorylation
Source: Sci Rep. 2020 Oct 9;10:16906. doi: 10.1038/s41598-020-73835-1 (PMC7547737; doi:10.1038/s41598-020-73835-1)
Supplement: Supplementary file 1 — Supplementary Information. [file 41598_2020_73835_MOESM1_ESM.docx]

**Supplementary INFORMATION** for

Direct stimulation of ERBB2 highlights a novel cytostatic signaling pathway driven by the receptor Thr^701^ phosphorylation

Marco Gaviraghi^§^, Andrea Rabellino^#,§^, Annapaola Andolfo, Matthias Brand^¶^, Chiara Brombin, Paola Bagnato, Giuseppina De Feudis, Andrea Raimondi, Alberta Locatelli, Daniela Tosoni, Davide Mazza, Luca Gianni, Giovanni Tonon, Yosef Yarden, Carlo Tacchetti^*^, Tiziana Daniele^*^

^1)^ Division of Experimental Oncology, Istituto di Ricovero e Cura a Carattere Scientifico (IRCCS) San Raffaele Scientific Institute, via Olgettina 60, 20132 Milan, Italy

^2)^ Department of Experimental Medicine, University of Genoa, via De Toni 14, 16132 Genoa, Italy

^3)^ Protein Microsequencing Facility, Istituto di Ricovero e Cura a Carattere Scientifico (IRCCS) San Raffaele Scientific Institute, via Olgettina 60, 20132 Milan, Italy

^4)^ Experimental Imaging Centre, Istituto di Ricovero e Cura a Carattere Scientifico (IRCCS) San Raffaele Scientific Institute, via Olgettina 58, 20132 Milan, Italy

^5)^ University Centre for Statistics in the Biomedical Sciences, Vita-Salute San Raffaele University, via Olgettina 58, 20132 Milan, Italy

^6)^ Department of Oncology, Istituto di Ricovero e Cura a Carattere Scientifico (IRCCS) San Raffaele Scientific Institute, via Olgettina 60, 20132 Milan, Italy

^7)^ European Institute of Oncology, 20100 Milan, Italy.

^8)^ Center for Translational Genomics and Bioinformatics, Istituto di Ricovero e Cura a Carattere Scientifico (IRCCS) San Raffaele Scientific Institute, via Olgettina 60, 20132 Milan, Italy

^9)^ Weizmann Institute of Science, Rehovot 76100, Israel

^10)^ Vita-Salute San Raffaele University, via Olgettina 58, 20132 Milan, Italy

^#^ Present address: QIMR Berghofer Medical Research Institute, Brisbane, Queensland 4029, Australia.

^¶^ Present address: CeMM Research Center for Molecular Medicine of the Austrian Academy of Sciences, A-1090 Vienna, Austria

^§^ These authors contributed equally to the work.

*****Equally contributing last and corresponding authors:

[daniele.tiziana@hsr.it](mailto:daniele.tiziana@hsr.it)

[tacchetti.carlo@hsr.it](mailto:tacchetti.carlo@hsr.it)

SUPPLEMENTARY Materials and Methods

SUPPLEMENTARY referenceS

SUPPLEMENTARY FIGURES

Supplementary Figure 1. TZ is effective in high ERBB2-expressing cells

Supplementary Figure 2. AKT downmodulation is an ERBB2-specific effect

Supplementary Figure 3. AKT downmodulation is an Ab-specific effect

Supplementary Figure 4. Abs trigger ERBB2 dimerization

Supplementary Figure 5. ERK-dependent AKT downmodulation does not involve pathways controlling AKT phosphorylation

Supplementary Figure 6. ERK-dependent phospho-Thr^308^ AKT downmodulation involves PP2A Ser/Thr phosphatases

Supplementary Figure 7. CyPA is not an AKT substrate and its silencing does not impair TZ signaling

Supplementary Figure 8. Images of full size/length gels/blots for Figure 1

Supplementary Figure 9. Images of full size/length gels/blots for Figure 2

Supplementary Figure 10. Images of full size/length gels/blots for Figure 3

Supplementary Figure 11. Images of full size/length gels/blots for Figure 4

Supplementary Figure 12. Images of full size/length gels/blots for Figure 5

Supplementary Figure 13. Images of full size/length gels/blots for Figure 6

Supplementary Figure 14. Images of full size/length gels/blots for Supplementary Figure 1

Supplementary Figure 15. Images of full size/length gels/blots for Supplementary Figure 2

Supplementary Figure 16. Images of full size/length gels/blots for Supplementary Figure 3

Supplementary Figure 17. Images of full size/length gels/blots for Supplementary Figure 4

Supplementary Figure 18. Images of full size/length gels/blots for Supplementary Figure 5

Supplementary Figure 19. Images of full size/length gels/blots for Supplementary Figure 6

Supplementary Figure 20. Images of full size/length gels/blots for Supplementary Figure 7

SUPPLEMENTARY TABLES

Supplementary Table S1: Mass spectrometry analysis of ERBB2 interactors

Supplementary Table S2: Mass spectrometry analysis of phospho-Thr^308^ AKT interactors

*SUPPLEMENTARY MATERIALS and METHODS*

Reagents and antibodies

All primary antibodies and HRP-conjugated secondary antibodies were purchased from Cell Signaling Technology unless otherwise stated. Antibodies specific for CyPA, total AKT, total ERK, PP2A C alpha subunit, HA-tag, and GAPDH were purchased from Abcam. Anti-N-terminal ERBB2 antibodies (9G6.10 and Ab-20), Mem-PE Plus Membrane Protein Extraction Kit, Fab Preparation Kit and BCA protein assay kit were purchased from Pierce (Thermo Scientific). Alexa 350, Alexa 488, Alexa 546, Alexa 568, and Alexa 647 secondary antibodies were obtained from Molecular Probes (Life Technologies, Thermo Scientific). Human, mouse and rabbit IgG (IP controls) were purchased from Jackson Research Laboratories. Control non-targeting (nt) small interfering oligonucleotides (siRNA), siRNA targeting EGFR and ERBB3, Lipofectamine 2000, Optimem, and Protein G and Protein A dynabeads were purchased from Life Technologies (Thermo Scientific). MTT, EGF, HRG, Glycine, saponin, NH_4_Cl, bovine serum albumin (BSA), phosphatase and protease inhibitors, FLAG-HRP antibody, and FLAG-beads were purchased from Sigma. Flag-tagged version of AKT1 wt was a gift from William Sellers (Addgene plasmid #9021) [1]. Flag-tagged version of CyPA wt was purchased from SinoBiological. All chemical reagents were of analytical grade or higher, and purchased from Sigma unless otherwise specified.

Cell culture and transfection

SK-Br3, BT474, MDA-MB-468, and MCF7 cell lines were purchased from American Type Culture Collection (ATCC).

Cells were grown in high-glucose (4500 mg/L) DMEM supplemented with 2 mM L-glutamine, 50 IU/mL penicillin, 50 mg/mL streptomycin and 10% foetal calf serum (FCS) at 37°C in 5% CO_2_ and at 98% humidity.

TZ and PZ were donated by the Pharmacy of the San Raffaele Hospital. TZ (dissolved at a stock concentration of 20 mg/ml with saline solution) was used at a working concentration of 10 µg/ml [2]. PZ (dissolved at a stock concentration of 30 mg/ml with saline solution) was used at a working concentration of 20 µg/ml [2].

Cells were treated with antibodies and/or 100 ng/ml EGF and/or 100 ng/ml HRG in DMEM without serum at 37°C.

To inhibit ERK phosphorylation, cells were pre-incubated with 10 µM U0126 (Promega) in serum-free DMEM at 37°C for 20 minutes, and maintained at the same concentration during the TZ treatment.

To inhibit EGFR and ERBB2 tyrosine kinase activity, cells were pre-incubated with 10 µM lapatinib (LC Laboratories), in serum-free DMEM at 37°C for 20 minutes, and maintained at the same concentration during the TZ treatment.

For IF experiments, SK-Br3 cells were harvested on glass coverslips the day before transfection. Cells were transfected using Lipofectamine 2000, accordingly to the manufacturer's instructions, and usually fixed 24h after transfection.

Clinical samples and cells

Fresh, frozen, mammary tissue specimens were collected at the European Institute of Oncology (IEO, Milan, Italy). All tissues were collected via standard operating procedures approved by the Institutional Ethical Board, and informed consent was obtained for all tissue specimens linked with clinical data.

To generate PDX models, fresh specimens from human BrCa were cut into 4 x 2 mm pieces using a razor blade, removing necrotic tissue, if present. The resulting fragments were embedded in ice-cold Matrigel (BD Matrigel, BD Biosciences) and immediately injected into the 4^th^ inguinal mammary glands of 8-week-old female NOD/SCID/IL2Rγ−/− (NSG) mice (Jackson Laboratories stock #5557) anesthetized by i.p. injection of 150 mg/kg tribromoethanol (Avertin). Animals were euthanized when the tumor exceeded 10% of the body mass in compliance with regulations for use of vertebrate animals in research. Bulk mammary epithelial cells (MECs) were isolated from human mammary tumour samples or from PDXs, and cultivated [7]. Briefly, tumour biopsies or PDXs were mechanically dissociated and enzymatically digested in DMEM/F12 medium supplemented with 1 mM glutamine, 200 U/ml collagenase (Sigma) and 100 U/ml hyaluronidase (Sigma) at 37°C for 4-5 h under rotating conditions. Single cell suspensions were obtained through an additional incubation of digested tissues in 0.05% trypsin-EDTA for 5 min at 37°C, plated on collagen-treated plates and used for the experiments.

RNA interference

To knockdown ERBB receptors, SK-Br3 cells were harvested the day before transfection (day 1). On days 2 and 3, cells were transfected using Lipofectamine 2000, according to the manufacturer's instructions, and 200 nM of either non-targeting (nt, Stealth RNAi siRNA Negative Control Low GC Duplex #2, Thermo Scientific) or EGFR- [HSS103116 EGFR (Human) Stealth siRNA Duplex, 5′-CCTATGCCTTAGCAGTCTTATCTAA-3′, Thermo Scientific] and ERBB3- [HSS140802 ERBB3 (Human) Stealth siRNA Duplex, 5′-GGCCATGAATGAATTCTCTACTCTA-3′, Thermo Scientific] specific siRNA pools. Cells were lysed on day 5.

EGFR protein levels were 100±21.5 in nt siRNA and 36.1±14.2 in EGFR+ERBB3 siRNA transfected cells. ERBB3 protein levels were 100±20.5 in nt siRNA and 34.6±10.1 in EGFR+ERBB3 siRNA transfected cells (out of four independent experiments).

To knockdown CyPA expression, a short hairpin sequence targeting human CyPA, retrieved from the Broad Institute TRC library database, was cloned into pLKO.1 lentiviral vector (Open Biosystems, Huntsville, AL, USA) and cells were infected. Non-targeting (nt) shRNA (low GC content) was used as a negative control.

The CyPA-specific oligonucleotide used was:

5′-CTGACTGTGGACAACTCGAAT-3′

The nt shRNA used was:

5'-CAACAAGATGAAGAGCACCAA-3'.

For viral production, each lentiviral plasmid DNA was co-transfected with pRSV-REV, pMD2 VSVG and pMDLg-pRRE plasmids into HEK-293T packaging cells. After 48 h, culture supernatants containing the released virus particles were filtered and used to infect SK-Br3 cells in combination with polybrene (10 µg/ml). The day after the infection, transduced SK-Br3 cells were splitted and then selected by 1.5 µM puromycin treatment for 14 days.

CyPA protein levels were 100±11.5 in SK-Br3 cells, 108.7±13.0 in nt shRNA, and 5.3±1.0 in CyPA-specific shRNA expressing cells.

Site-directed mutagenesis

AKT single mutants were generated with a single round of mutagenesis starting from a pCDNA3 Flag-HA AKT1 vector (Addgene plasmid # 9021, gift from William Sellers). AKT^P313A/P318A^ double mutant was obtained by performing a second mutagenic PCR starting from the AKT^P313A^-containing vector. The list of mutagenic primers is reported below:

| AKT T308A FWD | GGTGCCACCATGAAGGCCTTTTGCGGCACAC |
| --- | --- |
| AKT T308A REV | GTGTGCCGCAAAAGGCCTTCATGGTGGCACC |
| AKT T308D FWD | CGGTGCCACCATGAAGGACTTTTGCGGCACACCT |
| AKT T308D REV | AGGTGTGCCGCAAAAGTCCTTCATGGTGGCACCG |
| AKT P313A FWD | CCTTTTGCGGCACAGCTGAGTACCTGGCC |
| AKT P313A REV | GGCCAGGTACTCAGCTGTGCCGCAAAAGG |
| AKT P318A FWD | GAGTACCTGGCCGCCGAGGTGCTGG |
| AKT P318A REV | CCAGCACCTCGGCGGCCAGGTACTC |

ERBB2 single mutants were generated with a single round of mutagenesis starting from a pLenti C-MYC-DDK vector containing ERBB2 coding sequence fused in frame with GFP-tag downstream. The vector was amplified with specific mutagenic primers obtaining linear products that were digested with Dpn1 to remove the parental template, phosphorylated at their 5’ edge with T4 polynucleotide kinase and re-circularized by incubating with T4-DNA ligase. The list of mutagenic primers is reported below:

| ERBB2 P702A FWD | GCCGCTGACAGCTAGCGGAGC |
| --- | --- |
| ERBB2 P702A REV | TCCACCAGCTCCGTTTCCTG |

Sequences of mutated plasmids were verified by Sanger sequencing (GATC Biotech) using specific primers.

Immunofluorescence (IF)

Cells grown on glass coverslips were fixed in 4% paraformaldehyde and permeabilised with blocking solution (PBS supplemented with 0.1% saponin, 0.5% BSA and 50 mM NH_4_Cl). Cells were incubated with primary antibodies and with specific Alexa 350, 488 and/or 546-conjugated secondary antibodies diluted in blocking solution. For imaging, samples were examined using a Zeiss Imager A2 microscope, equipped with 49 DAPI (excitation 365, beam splitter FT 395, emission BP 445/50), 43 HE CY3 (excitation BP 550/25, beam splitter FT 570, emission BP 605/70) and 38 HE EGFP (excitation BP 470/40, beam splitter FT 495, emission BP 525/50) filter sets (Zeiss). Images were obtained under a 63x/1.40 oil-immersion Plan-Apochromat M27 objective (Zeiss), at a definition of 1388 x1040 pixels (150 dpi), by means of a high resolution monochromatic camera (Axiocam MRm Rev3, Zeiss), and analysed with the Axiovision REL 4.7 software (Zeiss).

Western blotting (WB)

Cells were washed twice with ice-cold PBS, scraped in lysis buffer (containing 20 mM Hepes, 150 mM NaCl, 1% Triton-X100 and 10% glycerol pH 7.4, complete protease inhibitor (Roche), phosphatase inhibitors (Sigma), 1 mM NaF, 1 mM sodium orthovanadate, and 1 mM beta-glycerophosphate), and incubated for 15 min on a wheel at 4°C. Supernatant was centrifuged at 16,000 g for 1 min at 4°C to remove nuclei and membranes. Protein concentrations were determined (BCA protein assay), and 15 μg of proteins were used for SDS–PAGE and western blotting. Samples were prepared by adding 2x sample buffer and boiling for 5 min at 95°C before loading onto the gel. Proteins were separated by SDS–PAGE and transferred onto nitrocellulose membranes (Hybond, GE Healthcare). Strips containing the proteins of interest were incubated in 5% (w/v) BSA in TBS containing 0.1% (v/v) Tween-20, pH 7.4 (T-TBS), for 1 h at room temperature and then with fresh blocking buffer containing the primary antibody at its working concentration. After 3 h incubation at room temperature, or overnight at 4°C, the antibodies were removed and the strips washed with T-TBS for 3x 10 min. Strips were incubated for 1 h with the appropriate horse radish peroxidase (HRP)-conjugated secondary antibody and washed 3x 10 min with T-TBS and once with TBS. Western blots were developed using the chemiluminescent method (ECL, GE Healthcare) and protein signals were detected using Amersham Hyperfilm ECL (GE Healthcare). When necessary, the antibody was removed by incubation in stripping buffer (62.5 mM Tris–HCl, pH 6.7; 2% sodium dodecyl sulphate and 100 mM b-mercaptoethanol) at 55°C for 30 min, followed by extensive washing. Filters were then probed with the peroxidase-conjugated secondary antibody and developed using ECL, before incubation with a new primary antibody. Bands were quantified by densitometric analysis using the National Institutes of Health (NIH) ImageJ program. The quantification of each band was normalized using the signal of GAPDH as a loading control. The quantification of TZ-treated samples was normalized for the signal of the corresponding control (starved cells and/or DMSO-treated starved cells, when TZ was given in combination with inhibitors). As for IP quantification, amounts of co-immunoprecipitated proteins were normalized both on the immunoprecipitated protein and on their levels in control untreated SKBr3 cells.

Statistical information

Data on the de-phosphorylation kinetics of AKT residues upon treatment with TZ either alone or in combination with inhibitors, over time (at 0, 2, 20, 45 minutes), were modeled by means of a Linear Mixed Effects model (LME [3]). Treatment indicator and time, along with their interaction, were included as covariates in the model. Differently from traditional linear models, LME models are suited for representing clustered, and therefore dependent, data arising when data are gathered over time on the same unit. In particular, random components can be specified in the model, thus allowing the evaluation of unobserved biological heterogeneity. In our case random intercept models were built. Moreover, repeated measures problems were reduced to the case of two or three independent sample tests at a fixed time point. In particular, for each time point, exact permutation t-tests with Monte Carlo resampling or Kruskal Wallis test were performed. In the latter case, post-hoc pairwise multiple comparisons between group levels, according to Dunn and Bonferroni correction of *p*-values were applied. LME and nonparametric permutation tests were fitted in R (version 3.2.2) by using the following packages: *nlme* [Pinheiro J, Bates D, DebRoy S, Sarkar D and R Core Team (2016). nlme: Linear and Nonlinear Mixed Effects Models. R package version 3.1-128, http://CRAN.R-project.org/package=nlme], *perm* [4] and *PMCMR* [Pohlert, T. (2014). The Pairwise Multiple Comparison of Mean Ranks Package (PMCMR). R package. http://CRAN.R-project.org/package=PMCMR]. For all the analyses, significance level α was set equal to 0.05.

*SUPPLEMENTARY REFERENCES*

1	Hsieh, A. C. et al. A library of siRNA duplexes targeting the phosphoinositide 3-kinase pathway: determinants of gene silencing for use in cell-based screens. *Nucleic Acids Res* **32**, 893-901 (2004).

2	Yeon, C. H. & Pegram, M. D. Anti-erbB-2 antibody trastuzumab in the treatment of HER2-amplified breast cancer. *Invest New Drugs* **23**, 391-409 (2005).

3	Laird, N. M. & Ware, J. H. Random-effects models for longitudinal data. *Biometrics* **38**, 963-974 (1982).

4	Fay, M. P. & Shaw, P. A. Exact and Asymptotic Weighted Logrank Tests for Interval Censored Data: The interval R package. *J Stat Softw* **36**, i02 (2010).

5	Lammers, M., Neumann, H., Chin, J. W. & James, L. C. Acetylation regulates cyclophilin A catalysis, immunosuppression and HIV isomerization. *Nat Chem Biol* **6**, 331-337 (2010).

6	Ishihama, Y. et al. Exponentially modified protein abundance index (emPAI) for estimation of absolute protein amount in proteomics by the number of sequenced peptides per protein. *Mol Cell Proteomics* **4**, 1265-1272 (2005).

7	Tosoni, D., Di Fiore, P.P., & Pece, S. Functional purification of human and mouse mammary stem cells. *Methods Mol Biol* **916**, 59-79 (2012).

*Supplementary figureS*

**Supplementary Figure 1: TZ is effective in high-ERBB2 expressing cells.** (**a**) Lysates of MDA-MB-468, MCF7, BT474, SK-Br3 (SK-Br3 wt) and SK-Br3 grown in the continuous presence of 200 μg/ml TZ (SK-Br3R) cells were analysed by WB. Arrow indicates ERBB4 specific band. (**b-c**) Cells derived from immortalized cell lines expressing different levels of ERBB2 (BT474 and MDA-MB-468, **b**) or a PDX developed from an ERBB2-positive patient (**c**) were serum-starved for 20 min in the presence of 10 μM U0126 or vehicle (DMSO), and treated with 10 μg/ml TZ at 37°C for the indicated times. Lysates were analysed by WB. Arrow indicates ERBB2 specific band. Only 18% of the cells obtained from the PDX developed from an ERBB2-positive patient were ERBB2-positive, as evaluated by IF. The images of the full scan western blots are provided in Supplementary Fig. 14.

**Supplementary Figure 2**: **AKT downmodulation is an ERBB2-specific effect**. (**a**) SK-Br3 cells were transfected with control non-targeting (nt), EGFR-specific and ERBB3-specific, or EGFR-specific or ERBB3-specific siRNAs for 72 h. Lysates were analysed by WB. (**b**) SK-Br3 cells were transfected with EGFR-specific and ERBB3-specific, or control non-targeting (nt) siRNAs for 72 h. Cells were serum-starved for 20 min in the presence of 10 μM U0126 or vehicle (DMSO), and treated with 20 μg/ml PZ or 10 μg/ml TZ at 37°C for the indicated times. Lysates were analysed by WB. One experiment is shown as representative of three. Samples treated with each Ab in the presence of DMSO, or U0126 derive from the same experiment and the gels/blots have been processed in parallel. The images of the full scan western blots are provided in Supplementary Fig. 15.

**Supplementary Figure 3**: **AKT downmodulation is an Ab-specific effect.** (**a-c**) SK-Br3 cells were serum-starved for 20 min and treated with 10 μg/ml TZ or 20 μg/ml PZ (**a**) or 100 ng/ml EGF (**b**) or 100 ng/ml heregulin β1 (HRG, **c**) at 37°C for the indicated times. Lysates were analysed by WB. One experiment is shown as representative of two. (**d**) Cells were serum-starved for 20 min in the presence of 10 μM lapatinib or vehicle (DMSO), and treated with 10 μg/ml TZ or 20 μg/ml PZ at 37°C for the indicated times. Lysates were analysed by WB. One experiment is shown as representative of two. The images of the full scan western blots are provided in Supplementary Fig. 16.

**Supplementary Figure 4: Abs trigger ERBB2 dimerization.** SK-Br3 cells were serum-starved for 20 min at 37°C, treated with 10 μg/ml TZ or TZ-Fab or with 20 μg/ml PZ or PZ-Fab for 10 min at 37°C, and cross-linked with cell impermeable DTSSP for additional 10 min at room temperature. (**a-d**) Lysates were immunoprecipitated with an ERBB2-specific antibody (starved) and/or an anti-human specific antibody (all samples). Immunoprecipitates were subjected to non-reducing SDS-PAGE, and Coomassie staining (**a-b**) followed by mass spectrometry or WB analysis (**d**). (**c**) Lysates (input) and IP unbound fractions were subjected to reducing SDS-PAGE, and analysed by WB. The EGFR and vinculin blots were cropped from the same gel. The ERBB2 and vinculin blots were cropped from the same gel. Percentages indicate residual ERBB2 after immunoprecipitation. The images of the full scan gels and western blots are provided in Supplementary Fig. 17.

**Supplementary Figure 5**: **ERK-dependent AKT downmodulation does not involve pathways controlling AKT phosphorylation.** (**a-b**) Schematic representation of AKT phosphorylation control by mTORC1 [A, modified from Song *et al*] and PTEN [B, modified from Zmajkovicova *et al*]. (**c-d**) SK-Br3 cells were serum-starved for 20 min in the presence of the MEK inhibitor U0126 or vehicle (DMSO), and treated with 10 μg/ml TZ at 37°C for the indicated times. (**c**) Lysates were analysed by WB. One experiment is shown, as representative of three. The p-Ser^473^ AKT and p-ERK blots were cropped from the same gel. Membranes were initially labelled with the anti-phospho Abs, stripped and afterwards probed with the anti-total Abs. Samples treated with TZ in the presence of DMSO, or U0126 derive from the same experiment and the gels/blots have been processed in parallel. (**d**) Cytosolic (cyt) and membrane (mem) fractions were analysed by WB. One experiment is shown, as representative of three. The ERBB2, p-Ser^473^ AKT, p-ERK and GAPDH blots were cropped from the same gel. The MAGI1, p-PTEN and p-MEK1 blots were cropped from the same gel. Membranes were initially labelled with the anti-phospho Abs, stripped and afterwards probed with the anti-total Abs. Samples treated with TZ in the presence of DMSO, or U0126 derive from the same experiment and the gels/blots have been processed in parallel. MAGI1 doublet is indicated by square brackets. (**e**) SK-Br3 cells were serum-starved for 20 min in the presence of 10 μM U0126 (MEK1 inhibitor), or 10 μM LY294002 (PI3K inhibitor) or 20 nM rapamycin (mTORC1 inhibitor), or vehicle (DMSO), and treated with 10 μg/ml TZ at 37°C for the indicated times. Lysates were analysed by WB. One experiment is shown as representative of two. The p-S6kinase and p-ERK blots were cropped from the same gel. The p-Thr^308^ AKT and GAPDH blots were cropped from the same gel. Samples treated with TZ in the presence of DMSO, or U0126 or LY294002 or rapamycin derive from the same experiment and the gels/blots have been processed in parallel. Arrow indicates p70S6 kinase (the antibody also recognizes p85, which is not regulated by PI3K and mTORC1). The images of the full scan western blots are provided in Supplementary Fig. 18.

**Supplementary Figure 6: ERK-dependent phospho-Thr^308^ AKT downmodulation involves PP2A Ser/Thr phosphatases.** SK-Br3 cells were serum-starved for 20 min in the presence of the MEK inhibitor U0126 (10 μM, blue), or of a combination of tautomycin (5 nM) and okadaic acid (200 nM, red, or 400 nM, green), or vehicle (DMSO, black), and treated with 10 μg/ml TZ at 37°C for the indicated times. (**a**) Lysates were analysed by WB. One experiment is shown as representative of ten. The p-Ser^473^ AKT and p-ERK blots were cropped from the same gel. The p-Thr^308^ AKT and GAPDH blots were cropped from the same gel. Samples treated with TZ in the presence of DMSO, or U0126 or a combination of tautomycin (5 nM) and okadaic acid (200 nM or 400 nM) derive from the same experiment and the gels/blots have been processed in parallel. The images of the full scan western blots are provided in Supplementary Fig. 19. (**b-c**) Graphs show mean phospho-AKT levels for the Thr^308^ (**b**) and Ser^473^ (**c**) residue, out of ten independent experiments. Statistical analysis shows that Thr^308^ AKT residue is significantly more phosphorylated in the presence of higher concentrations of okadaic acid (p=0.031, out of ten independent experiments, linear mixed effects model) and that both Thr^308^ and Ser^473^ AKT residues are significantly more phosphorylated after 20 and 45 min of TZ/U0126 treatment, as compared to TZ treatment (p<0.0001, linear mixed effects model).

**Supplementary Figure 7: CyPA is not an AKT substrate and its silencing does not impair TZ signaling.** (**a**) SK-Br3 cells were serum-starved for 20 min and incubated with 10 μg/ml TZ at 37°C for 20 minutes in the presence of 10 μM U0126 or vehicle (DMSO). Cells were immunoprecipitated with an antibody specific for CyPA or with mouse IgG, as control. Immunoprecipitates were analysed by WB. Acetyl-lysine immuno-labelling is shown as a proof of principle that we could detect CyPA post-translational modifications [5]. (**b**) SK-Br3 cells were either not transduced or transduced with a non-targeting (nt) or a CyPA-specific shRNA for 15 days. Cells were serum-starved for 20 min in the presence of the MEK inhibitor U0126, and treated with 10 μg/ml TZ at 37°C for the indicated times. Data points from five independent experiments are colour-coded. A statistically significant difference was found at 20 and 45 min of treatment upon comparison of CyPA-silenced with control SK-Br3 cells (Kruskal Wallis and post-hoc test with Bonferroni correction, *p*=0.026 and *p*=0.014, respectively). (**c**) SK-Br3 cells were either left untreated or transduced with a non-targeting (nt) shRNA or a CyPA-specific shRNA for 15 days. Cells were serum-starved for 20 min, and treated with 10 μg/ml TZ at 37°C for the indicated times. Lysates were analysed by WB. One experiment is shown as representative of five. The not transduced (SK-Br3), control (nt) shRNA- or CyPA-specific (CyPA) shRNA- transduced samples derive from the same experiment and the gels/blots have been processed in parallel. The images of the full scan western blots are provided in Supplementary Fig. 20.

**Supplementary Figure 8:** Images of full length blots of western analysis performed on SK-Br3 cells serum-starved, in the presence of 10 μM U0126 or vehicle (DMSO), and treated with 20 μg/ml Pertuzumab (PZ) or 10 μg/ml Trastuzumab (TZ) presented in the Figure 1a.

**Supplementary Figure 9:** Images of the full length blots of western analysis performed on SK-Br3 cells either left untreated or transduced with a non-targeting (nt) shRNA or a CyPA-specific shRNA, serum-starved in the presence of U0126, and treated with 10 μg/ml Trastuzumab (TZ) presented in the Figure 2b.

**Supplementary Figure 10:** Images of the full length blots of western analysis performed on the immunoprecipitates (with a p-Thr^308^ AKT-specific antibody or with rabbit IgG, as control) of SK-Br3 cells either left untreated or serum-starved in the presence of U0126 or vehicle, and treated with 10 μg/ml Trastuzumab (TZ) presented in the Figure 3a.

**Supplementary Figure 10:** Images of the full length blots of western analysis performed on the immunoprecipitates (with a CyPA-specific antibody or with mouse IgG, as control) of SK-Br3 cells either left untreated or serum-starved in the presence of U0126 or vehicle, and treated with 10 μg/ml Trastuzumab (TZ) presented in the Figure 3b.

**Supplementary Figure 11:** Images of the full length blots of western analysis performed on the immunoprecipitates (with an antibody specific for the FLAG tag) of SK-Br3 cells expressing FLAG-tagged AKT Thr mutants or wt protein, serum-starved in the presence of U0126, and treated with 10 μg/ml Trastuzumab (TZ) presented in the Figure 4a.

**Supplementary Figure 11:** Images of the full length blots of western analysis performed on the immunoprecipitates (with an antibody specific for the FLAG tag) of SK-Br3 cells expressing FLAG-tagged AKT Pro mutants or wt protein, serum-starved in the presence of U0126, and treated with 10 μg/ml Trastuzumab (TZ) presented in the Figure 4a.

**Supplementary Figure 11:** Images of the full length blots of western analysis performed on the immunoprecipitates (with a CyPA-specific antibody or with mouse IgG, as control) of SK-Br3 cells expressing FLAG-tagged wt or T308A mutant AKT serum-starved, and treated with 10 μg/ml Trastuzumab (TZ) presented in the Figure 4b.

**Supplementary Figure 12:** Images of the full length blots of western analysis performed on the immunoprecipitates (with an ERBB2-specific antibody) of SK-Br3 cells serum-starved, and treated with 10 μg/ml Trastuzumab (TZ) presented in the Figure 5a.

**Supplementary Figure 12:** Images of the full length blots of western analysis performed on the immunoprecipitates (with an ERBB2-specific antibody) of SK-Br3 cells serum-starved, and treated with 20 μg/ml Pertuzumab (PZ) presented in the Figure 5a.

**Supplementary Figure 12:** Images of the full length blots of western analysis performed on SK-Br3 cells either left untreated or serum-starved in the presence of U0126 or vehicle, and treated with 10 μg/ml Trastuzumab (TZ) or 20 μg/ml Pertuzumab (PZ) presented in the Figure 5c.

**Supplementary Figure 13:** Images of the full length blots of western analysis performed on SK-Br3 cells either left untreated or transduced with a non-targeting (NT) shRNA or a CyPA-specific shRNA presented in the Figure 6a.

**Supplementary Figure 13:** Images of the full length blots of western analysis performed on the immunoprecipitates (with an antibody specific for the GFP tag) of SK-Br3 cells expressing GFP-tagged ERBB2 P702A mutant or wt protein serum-starved, and treated with 10 μg/ml Trastuzumab (TZ) presented in the Figure 6c.

**Supplementary Figure 13:** Images of the full length blots of western analysis performed on the immunoprecipitates (with an antibody specific for the GFP tag) of SK-Br3 cells expressing GFP-tagged ERBB2 P702A mutant or wt protein serum-starved, and treated with 20 μg/ml Pertuzumab (PZ) presented in the Figure 6d.

**Supplementary Figure 14:** Images of the full size blots of western analysis performed on MDA-MB-468, MCF7, BT474, and SK-Br3 cell lysates presented in the Supplementary Figure 1A.

**Supplementary Figure 14:** Images of full length blots of western analysis performed on BT474 and MDA-MB-468 (468) cells serum-starved, in the presence of 10 μM U0126 or vehicle (DMSO), and treated with 10 μg/ml Trastuzumab (TZ) presented in the Supplemetary Figure 1b.

**Supplementary Figure 14:** Images of full length blots of western analysis performed on cells from a PDX serum-starved, in the presence of 10 μM U0126 or vehicle (DMSO), and treated with 10 μg/ml Trastuzumab (TZ) presented in the Supplementary Figure 1c.

**Supplementary Figure 15:** Images of the full size blots of western analysis performed on SK-Br3 cells transfected with control non-targeting (nt), EGFR-specific and ERBB3-specific, or EGFR-specific or ERBB3-specific siRNAs presented in the Supplementary Figure 2a.

**Supplementary Figure 15:** Images of the full size blots of western analysis performed on SK-Br3 cells transfected with EGFR-specific and ERBB3-specific, or control non-targeting (nt) siRNAs, serum-starved in the presence of U0126 or vehicle (DMSO), and treated with 20 μg/ml PZ or 10 μg/ml TZ presented in the Supplementary Figure 2b.

**Supplementary Figure 16:** Images of the full size blots of western analysis performed on SK-Br3 cells serum-starved and treated with 10 mg/ml TZ presented in the Supplementary Figure 3a.

**Supplementary Figure 16:** Images of the full size blots of western analysis performed on SK-Br3 cells serum-starved and treated with 20 mg/ml PZ presented in the Supplementary Figure 3a.

**Supplementary Figure 16:** Images of the full size blots of western analysis performed on SK-Br3 cells serum-starved and treated with 100 ng/ml EGF presented in the Supplementary Figure 3b.

**Supplementary Figure 16:** Images of the full size blots of western analysis performed on SK-Br3 cells serum-starved and treated with 100 ng/ml Heregulin b1 (HRG) presented in the Supplementary Figure 3c.

**Supplementary Figure 16:** Images of the full size blots of western analysis performed on SK-Br3 cells serum-starved in the presence of lapatinib (10 μM), or vehicle (DMSO) and treated with 10 mg/ml TZ presented in the Supplementary Figure 3d.

**Supplementary Figure 16:** Images of the full size blots of western analysis performed on SK-Br3 cells serum-starved in the presence of lapatinib (10 μM), or vehicle (DMSO) and treated with 20 mg/ml PZ presented in the Supplementary Figure 3d.

**Supplementary Figure 17:** Images of the full size gels and blots of western analysis performed on SK-Br3 cells serum-starved, treated with 10 μg/ml TZ or TZ-Fab or with 20 μg/ml PZ or PZ-Fab, cross-linked with cell impermeable DTSSP and immunoprecipitated with an ERBB2-specific antibody (starved) and/or an anti-human specific antibody (all samples) presented in the Supplementary Fig. 4.

**Supplementary Figure 18:** Images of the full size blots of western analysis performed on SK-Br3 cells serum-starved in the presence of the MEK inhibitor U0126 (10 μM), or vehicle (DMSO) and treated with 10 mg/ml TZ presented in the Supplementary Figure 5c.

**Supplementary Figure 18:** Images of full length blots of western analysis performed on the cytosolic (cyt) and membrane (mem) fractions of SK-Br3 cells serum-starved, in the presence of 10 μM U0126 or vehicle (DMSO), and treated with 10 μg/ml Trastuzumab (TZ) presented in the Supplementary Figure 5d.

**Supplementary Figure 18:** Images of the full size blots of western analysis performed on SK-Br3 cells serum-starved in the presence of the MEK inhibitor U0126 (10 μM), or LY294002 (10 μM) or rapamycin (20 nM), or vehicle (DMSO) and treated with 10 mg/ml TZ presented in the Supplementary Figure 5e.

**Supplementary Figure 19:** Images of the full size blots of western analysis performed on SK-Br3 cells serum-starved in the presence of the MEK inhibitor U0126 (10 μM), or of a combination of tautomycin (5 nM) and okadaic acid (200 nM or 400 nM), or vehicle (DMSO) and treated with 10 mg/ml TZ presented in the Supplementary Figure 6a.

**Supplementary Figure 20:** Images of the full length blots of western analysis performed on the immunoprecipitates (with a CyPA-specific antibody or with mouse IgG, as control) of SK-Br3 cells serum-starved in the presence of U0126 or vehicle, and treated with 10 μg/ml Trastuzumab (TZ) presented in the Supplementary Figure 7a.

**Supplementary Figure 20 (bottom):** Images of the full length blots of western analysis performed on SK-Br3 cells either left untreated or transduced with a non-targeting (nt) shRNA or a CyPA-specific shRNA, serum-starved in the presence of vehicle (DMSO), and treated with 10 μg/ml Trastuzumab (TZ) presented in the Supplementary Figure 7c.

*Supplementary TABLEs*

**Supplementary Table S1:** **Mass spectrometry analysis of ERBB2 interactors**

MaxQuant results are shown. The sheets report the identified and quantified protein groups along with their accession number ID, protein name, gene name, MW, sequence length, score and intensities for each protein group in three technical replicates. Searches were performed using the UniProt_Human Complete Proteome_cp_hum_20180228 (93,786 sequences; 37,179,059 residues). EGFR and ERBB2 data are colour-coded: red and blue, respectively.

**Supplementary Table S2:** **Mass spectrometry analysis of phospho-Thr^308^ AKT interactors**

Mascot results are shown. Each sheet reports the identified protein families, the corresponding protein members along with their accession number ID, Mascot score, MW, number of sequenced peptides, emPAI (exponentially modified protein abundance index), which is proportional to protein content in a protein mixture (as proposed by Ishihama et al [6]) and description. Searches were performed using the UniProt_Human Complete Proteome_cp_hum_20170315 (92,919 sequences; 36,868,442 residues).
